# Do serum vitamins, carotenoids, and retinyl esters influence mortality in osteoarthritis? Insights from a nationally representative study

**DOI:** 10.3389/fnut.2025.1609759

**Published:** 2025-06-19

**Authors:** Yifan Lu, Junjie Duan, Rong You, Simin Tang, Xinyu Qi, Boyu Wu, Gonghui Jian, Tao Wang, Jianhui Duan, Zhuo Yang

**Affiliations:** ^1^Department of Orthopedics, Changde First Hospital of Traditional Chinese Medicine, Changde, Hunan, China; ^2^Department of Graduate School, Hunan University of Traditional Chinese Medicine, Changsha, Hunan, China; ^3^Department of Orthopedics, Dongguan Hospital of Guangzhou University of Chinese Medicine, Dongguan, Guangzhou, China

**Keywords:** osteoarthritis, serum vitamins, carotenoids, mortality, NHANES

## Abstract

**Background:**

The relationship between serum vitamins, carotenoids, and retinyl esters and mortality risk among individuals with osteoarthritis (OA) remains unclear. This study aimed to investigate these associations.

**Methods:**

Based on data from NHANES 2001 to 2018 and the National Death Index (NDI), a total of 3,626 patients with OA were included. Cox proportional hazards models were used to evaluate the associations between serum nutrient levels and all-cause, cardiovascular, and cancer mortality. Nonlinear effects were assessed using smooth curve fitting and piecewise regression models. Subgroup analyses, sensitivity analyses, and validation in non-OA populations were conducted. Additionally, interactions between vitamin levels and OA status were examined.

**Results:**

Higher levels of vitamin D, retinyl palmitate, and stearic acid were associated with a reduced risk of all-cause mortality in patients with OA, with consistent results in sensitivity analyses. A nonlinear inverse association was observed between vitamin D and all-cause mortality in women, with a threshold at 30.5 nmol/L. Vitamin C was associated with cardiovascular mortality, while retinyl palmitate and stearic acid were linked to a reduced risk of cancer-related death. The protective effect of vitamin D was stronger among individuals with lower educational levels. In the non-OA population, only vitamin D was associated with mortality. Interaction analysis indicated that high vitamin levels may attenuate the adverse impact of OA on mortality risk.

**Conclusions:**

Elevated serum levels of vitamin D, retinyl palmitate, and stearic acid may be associated with reduced all-cause and cause-specific mortality among individuals with OA, highlighting their potential role in the management of OA.

## 1 Introduction

Osteoarthritis (OA), a chronic joint disorder characterized by progressive cartilage degeneration, significantly impairs the quality of life and functional status among middle-aged and older adults worldwide (1, 2). Since the beginning of the 21st century, the global burden of OA has continued to rise in parallel with population growth and accelerated aging. This trend has also been accompanied by disparities in regional case distributions and disease management capacities (3, 4). Previous studies have predominantly focused on the disabling effects and quality-of-life consequences of OA (5, 6), while limited attention has been paid to the role of nutritional strategies in the long-term prognostic management of OA, representing a crucial gap in the literature.

Serum vitamins, carotenoids, and retinyl esters, as essential antioxidant micronutrients, are actively involved in immune regulation, inflammation control, and cellular homeostasis (7–9). Emerging evidence suggests that folate, vitamin B12, and vitamin D may play critical roles in the progression and clinical outcomes of OA (10–13). Importantly, the intake and availability of these nutrients are relatively less influenced by socioeconomic disparities, implying that adequate nutritional status could be achievable even in resource-limited settings. Therefore, identifying modifiable, nutrition-related factors may offer new perspectives for the long-term management of OA, especially in underserved populations.

In recent years, a growing number of studies have explored the associations between serum vitamins, carotenoids, and retinyl esters and mortality risks in various health conditions, including SARS-CoV-2 infection, prediabetes, and depression (14–16). However, the potential impact of these micronutrients on mortality risk among individuals with OA remains largely unexplored. Based on these findings, this study systematically explored the associations between various serum vitamins, carotenoids, and vitamin A esters and mortality risk in patients with OA. Through multi-level analyses, the robustness and specificity of the results were validated, aiming to provide epidemiological evidence for precision nutritional interventions and long-term health management in the OA population.

## 2 Methods

### 2.1 Study population

**Figure 1:**
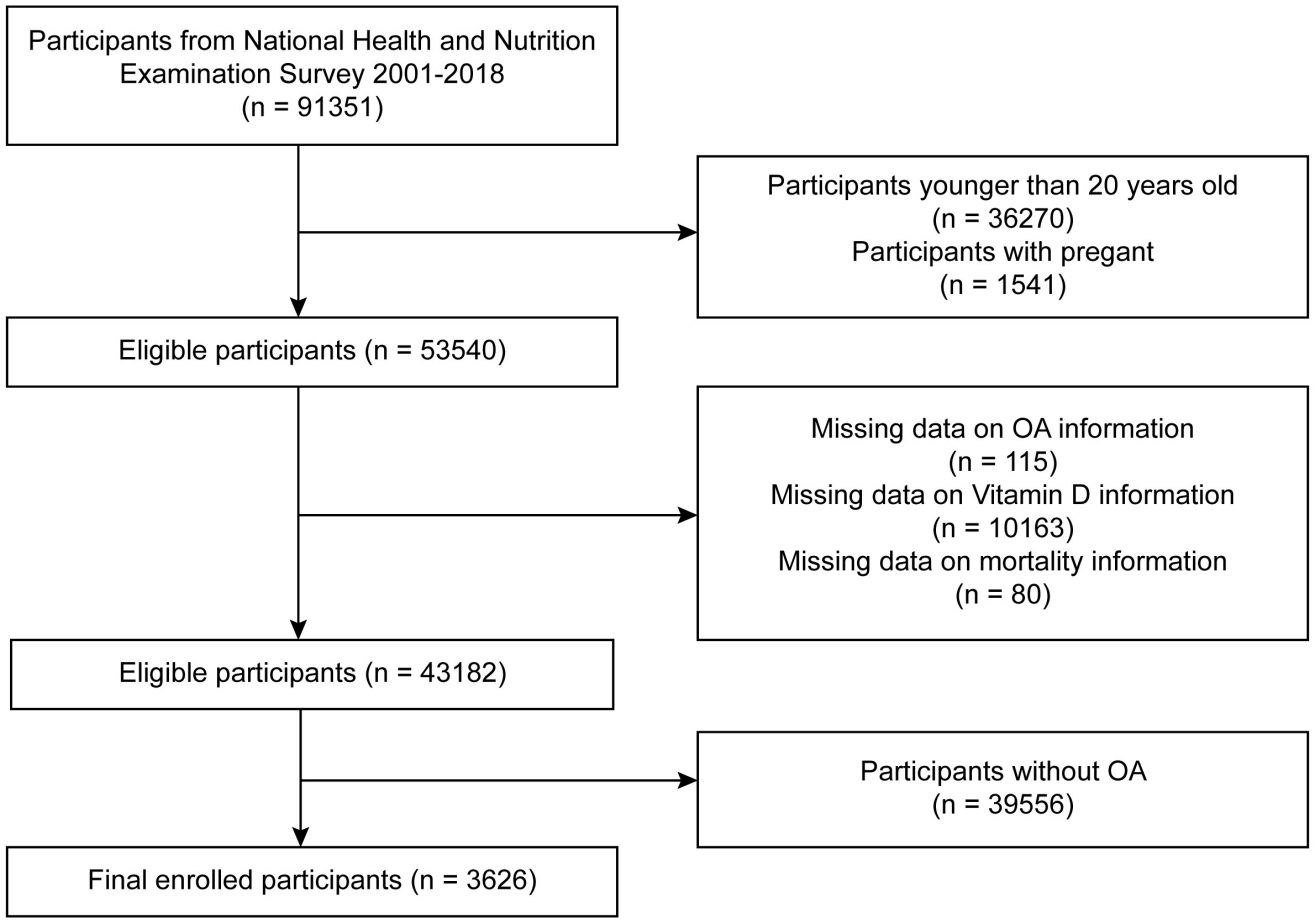
Flow chart (vitamin D).

This study was based on publicly available data from the NHANES, which employs a stratified multistage sampling design to represent the non-institutionalized U.S. population. We utilized data from nine consecutive NHANES cycles spanning from 2001 to 2018 (each cycle representing a 2-year interval). A total of 91,351 participants were initially identified. We sequentially excluded individuals who met the following criteria: age <20 years (*n* = 36,270), pregnant women (*n* = 1,541), missing OA status (*n* = 115), missing serum vitamin D data (*n* = 10,163), missing mortality follow-up information (*n* = 80), and individuals without OA (*n* = 39,556). The final analytic sample included 3,626 participants with diagnosed OA (Figure 1). Participants with missing serum vitamin D data were excluded because vitamin D was the main exposure variable. To avoid potential bias, we did not use multiple imputation for this variable, as imputing primary exposures with non-random missingness may introduce error. This is a common approach in epidemiologic studies focused on exposure-outcome associations. To maximize the use of available data, individual datasets were separately cleaned for each specific vitamin or carotenoid variable. Detailed procedures are illustrated in Supplementary Figures 1A, B.

### 2.2 Definition of study variables

Data on serum vitamins (C, D, and E), vitamin A, and its related compounds—including carotenoids (α-carotene, trans-β-carotene, cis-β-carotene, β-cryptoxanthin, lutein/zeaxanthin, and trans-lycopene) and vitamin A esters (retinyl palmitate and retinyl stearate)—were obtained from laboratory measurements in NHANES. These biomarkers were quantified using high-performance liquid chromatography (HPLC), and they provide an objective assessment of individuals' nutritional status. OA status was determined based on the Medical Conditions Questionnaire (MCQ) module in NHANES. Participants were classified as having OA if they answered “Yes” to the question, “Doctor ever said you had Osteoarthritis.” Those who answered “No” were defined as non-OA participants, indicating that they had not been diagnosed with OA by a healthcare professional. The questionnaire was administered by trained interviewers in participants' homes using a computer-assisted personal interviewing (CAPI) system, which included built-in consistency checks and terminology prompts to improve data quality and minimize interview bias.

Covariates included in the analysis were as follows: (1) Demographic variables: age, gender, race/ethnicity, education level, marital status, and the poverty-income ratio (PIR), categorized as ≤ 1.3 (low), ≤ 3.5 (middle), and >3.5 (high), all derived from NHANES demographic datasets. (2) Anthropometric and laboratory variables: alanine aminotransferase (ALT), aspartate aminotransferase (AST), body mass index (BMI), and waist circumference. (3) Comorbidities and lifestyle factors: hypertension, diabetes, cardiovascular disease (CVD), smoking status, and drinking status. The detailed definitions and diagnostic criteria for these variables are provided in Supplementary material 1.

### 2.3 Mortality data

Mortality data were obtained through the linkage between NHANES and the NDI, with follow-up through December 31, 2019. Deaths were identified by matching population variables, and causes of death were classified according to ICD-10 codes. Participants without a matching record were considered alive. Among the included OA participants, there were a total of 734 all-cause deaths, including 202 deaths due to cardiovascular disease and 165 cancer-related deaths. As the database did not provide information on specific cancer types, cancer mortality was broadly defined and encompassed all deaths attributable to malignant neoplasms.

### 2.4 Statistical analysis

The analysis accounted for the complex sampling design of NHANES by using weighted methods to enhance representativeness. Continuous variables were presented as weighted means with 95% confidence intervals (CIs), and comparisons between groups were conducted using weighted linear regression. Categorical variables were expressed as weighted percentages and compared using the chi-square test. Kaplan–Meier curves and three Cox proportional hazards models were used to assess the associations between serum nutrients and mortality risk. Nonlinear relationships were examined using smooth curve fitting and segmented Cox models, with threshold effects determined by log-likelihood ratio tests. Stratified and interaction analyses were performed, and sensitivity analyses included excluding participants who died within the first 2 years of follow-up and those with baseline preCVD. The main analyses were replicated in the non-OA population, and interaction effects were further explored by grouping participants according to nutrient levels and OA status. All analyses were conducted using R version 4.2.0 and EmpowerStats version 2.0, with a two-sided *P*-value < 0.05 considered statistically significant.

## 3 Results

### 3.1 Baseline characteristics of participants with OA

Gender-stratified analysis of baseline characteristics among participants with OA revealed significant differences across multiple anthropometric and biochemical indicators. Male participants exhibited significantly higher waist circumference, ALT and AST levels compared to females. In contrast, female participants had notably higher serum levels of vitamins A, C, D, and E, as well as several carotenoids, including α-carotene, trans-β-carotene, cis-β-carotene, and lutein + zeaxanthin (Table 1).

**Table 1:** Baseline characteristics of the OA patient population based on gender.

**Variables**	**Female**	**Male**	***P*-value**
**For continuous variables, mean (95% CI)**			
Age (years)	60.70 (58.93–62.48)	58.86 (57.35–60.36)	0.1547
BMI (kg/m^2^)	30.76 (29.41–32.11)	30.28 (29.29–31.28)	0.6122
Waist circumference (cm)	100.99 (98.28–103.70)	107.55 (105.29–109.82)	0.0012
ALT (U/L)	20.07 (18.82–21.32)	28.35 (25.29–31.41)	<0.0001
AST (U/L)	21.63 (20.86–22.41)	26.30 (23.97–28.63)	0.0013
Vitamin A (μg/dl)	59.22 (57.66–60.78)	65.44 (62.65–68.22)	0.0002
Vitamin E (μg/dl)	1,454.18 (1,388.00–1,520.37)	1,314.17 (1,251.29–1,377.06)	0.0021
α-carotene (μg/dl)	4.47 (3.81–5.12)	3.16 (2.66–3.67)	0.0030
Trans-β carotene (μg/dl)	21.48 (17.74–25.21)	14.93 (12.27–17.58)	0.0081
Cis-β carotene (μg/dl)	1.27 (1.07–1.46)	0.93 (0.76–1.09)	0.0176
β-Cryptoxanthin (μg/dl)	8.13 (7.20–9.06)	7.42 (6.49–8.35)	0.1832
Lutein and zeaxanthin (μg/dl)	17.75 (16.20–19.31)	16.08 (14.66–17.50)	0.1462
Trans-lycopene (μg/dl)	19.36 (18.24–20.48)	20.26 (18.91–21.62)	0.2801
Retinyl palmitate (μg/dl)	1.94 (1.60–2.27)	1.87 (1.66–2.08)	0.7006
Retinyl stearate (μg/dl)	0.62 (0.54–0.70)	0.60 (0.54–0.65)	0.6476
**For categorical variables, percentage (95% CI)**			
**Race**			0.0659
Other Race—Including Multi-Racial	6.59 (4.32–9.93)	4.39 (2.65–7.19)	
Mexican American	4.79 (3.50–6.53)	3.30 (1.90–5.67)	
Other Hispanic	3.74 (2.32–5.99)	2.86 (1.27–6.34)	
Non-Hispanic White	71.36 (65.80–76.35)	79.70 (72.92–85.13)	
Non-Hispanic Black	13.51 (10.23–17.64)	9.74 (7.03–13.36)	
**Education level**			0.0137
College graduate or above	16.75 (12.96–21.36)	26.73 (20.60–33.90)	
Less than 9th grade	5.90 (4.24–8.16)	6.78 (4.74–9.62)	
9–11th grade (includes 12th grade with no diploma)	11.83 (9.46–14.69)	12.75 (9.18–17.44)	
High school graduate/GED or equivalent	29.41 (24.17–35.27)	24.74 (19.62–30.70)	
Some college or AA degree	36.11 (29.97–42.74)	29.00 (23.49–35.20)	
**PIR**			0.1088
Low	26.24 (21.67–31.38)	21.18 (16.31–27.04)	
Middle	35.81 (30.48–41.51)	33.05 (26.30–40.58)	
High	37.95 (32.10–44.18)	45.77 (36.58–55.25)	
**Marital status**			0.0028
Never married	6.74 (3.22–13.56)	7.14 (3.72–13.25)	
Married	52.99 (46.84–59.04)	63.32 (54.96–70.95)	
Widowed	21.86 (17.02–27.62)	7.48 (4.54–12.08)	
Divorced	13.29 (9.88–17.63)	14.76 (9.82–21.60)	
Separated	2.37 (1.51–3.69)	1.59 (0.78–3.21)	
Living with partner	2.75 (1.68–4.48)	5.71 (3.06–10.42)	
**Hypertension**			0.6131
No	48.08 (41.67–54.56)	45.98 (38.79–53.34)	
Yes	51.92 (45.44–58.33)	54.02 (46.66–61.21)	
**Diabetes**			0.1816
No	80.00 (76.43–83.15)	76.92 (71.48–81.59)	
Yes	20.00 (16.85–23.57)	23.08 (18.41–28.52)	
**PreCVD**			0.2518
No	79.51 (75.79–82.79)	76.78 (72.04–80.93)	
Yes	20.49 (17.21–24.21)	23.22 (19.07–27.96)	
**Smoking status**			<0.0001
Never	53.80 (47.34–60.13)	26.94 (20.75–34.18)	
Former	26.12 (21.91–30.82)	43.23 (37.19–49.49)	
Now	20.09 (15.27–25.96)	29.83 (24.41–35.88)	
**Drinking status**			<0.0001
Never	16.62 (12.58–21.64)	5.39 (2.85–9.96)	
Former	20.49 (17.50–23.85)	16.63 (11.61–23.25)	
Mild	36.57 (30.59–42.99)	43.65 (35.84–51.80)	
Moderate	18.14 (14.65–22.25)	11.14 (7.03–17.21)	
Severe	8.18 (4.89–13.36)	23.18 (17.08–30.66)	

For continuous variables: survey-weighted mean (95% CI), *P*-value was by survey-weighted linear regression.

For categorical variables: survey-weighted percentage (95% CI), *P*-value was by survey-weighted Chi-square test.

Regarding sociodemographic factors, education level differed significantly between gender, with a higher proportion of males having attained post-secondary education. In terms of lifestyle and clinical characteristics, smoking status varied markedly: a much greater proportion of females reported never smoking, whereas the prevalence of “now” and “former” smokers was substantially higher among males. Similarly, significant gender differences were observed in alcohol consumption, with a higher proportion of heavy drinkers among males. No significant gender differences were observed for age, β-cryptoxanthin, trans-lycopene, retinyl palmitate, retinyl stearate, race/ethnicity, hypertension, diabetes, or cardiovascular disease history. Detailed baseline characteristics stratified by gender and serum vitamin C or vitamin D levels are presented in Supplementary Table S1.

### 3.2 Associations between serum vitamins, carotenoids, retinyl esters, and mortality risk in OA patients

**Figure 2:**
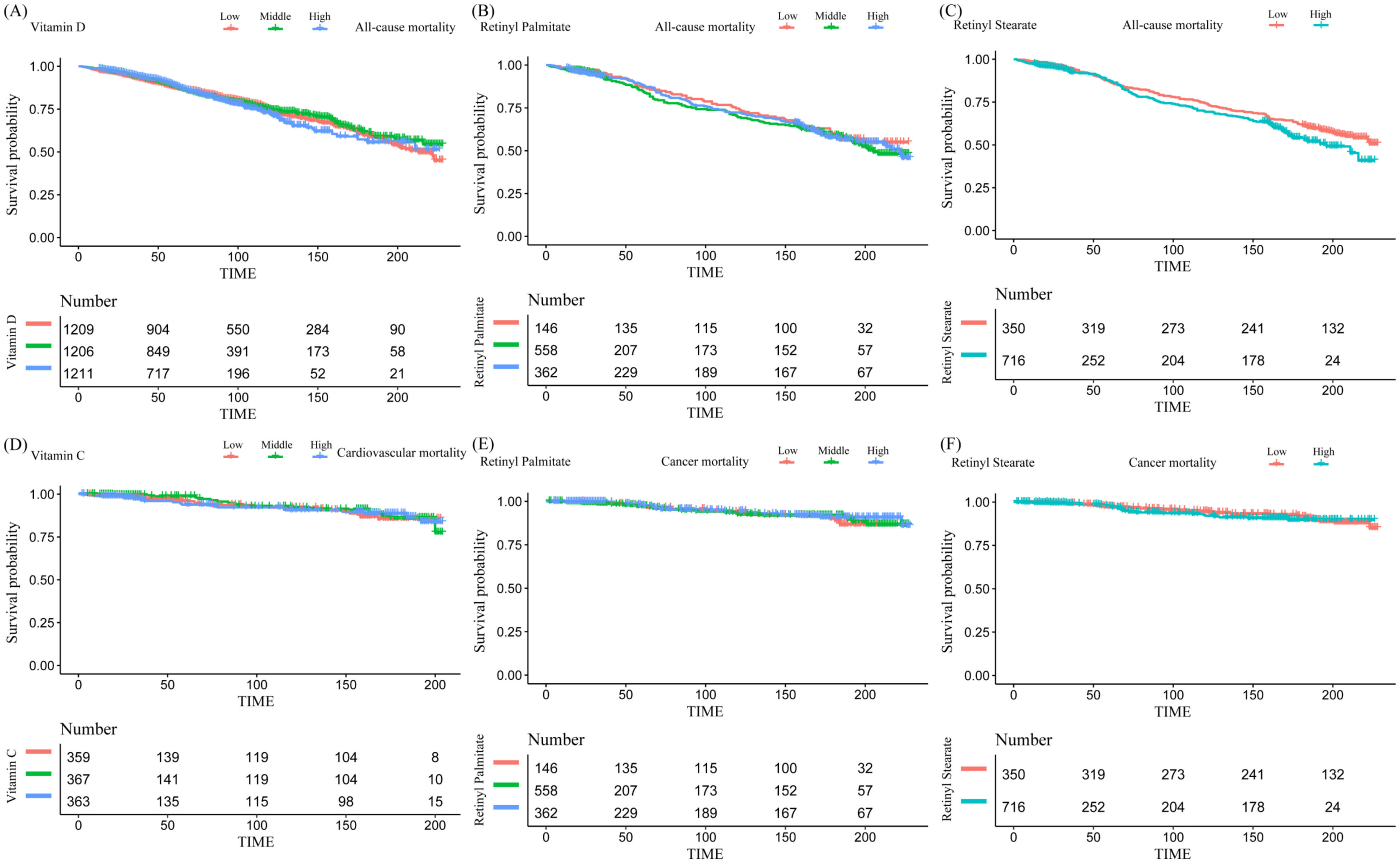
Kaplan–Meier curves for the associations between serum vitamin and retinyl ester levels and mortality risk in patients with osteoarthritis. **(A)** Vitamin D and all-cause mortality; **(B)** retinyl palmitate and all-cause mortality; **(C)** retinyl stearate and all-cause mortality; **(D)** vitamin C and cardiovascular mortality; **(E)** retinyl palmitate and cancer mortality; **(F)** retinyl stearate and cancer mortality.

Kaplan–Meier survival curves were generated to visually compare survival probabilities across exposure groups (Figure 2). Multivariable Cox proportional hazards regression models were then applied to examine the associations between serum nutrient levels and mortality risk, adjusting for a range of covariates (Table 2). In the analysis of all-cause mortality, Model 3 (fully adjusted) revealed that higher serum levels of vitamin D, retinyl palmitate, and retinyl stearate were significantly associated with reduced mortality risk.

**Table 2:** Cox regression analysis of serum vitamins, carotenoids, and retinyl esters in relation to mortality among osteoarthritis patients.

**Character**	**Model 1**		**Model 2**		**Model 3**	
	**HR (95%CI)**	***P*** **-value**	**HR (95%CI)**	***P*** **-value**	**HR (95%CI)**	***P*** **-value**
**All-cause mortality**						
Vitamin D	0.9978 (0.9942–1.0015)	0.2431	0.9914 (0.9875–0.9953)	<0.0001	0.994 (0.9902–0.9978)	0.0022
Retinyl palmitate	1.0261 (0.8995–1.1705)	0.7014	0.7647 (0.5861–0.9979)	0.0482	0.7186 (0.5622–0.9186)	0.0083
Retinyl stearate	0.9999 (0.9681–1.0327)	0.9936	0.9145 (0.8386–0.9974)	0.0435	0.9024 (0.8479–0.9604)	0.0012
**Cardiovascular disease mortality**						
Vitamin C	1.3365 (0.9009–1.9827)	0.1495	1.0735 (0.6131–1.8797)	0.8039	1.575 (1.0335–2.4002)	0.0346
**Cancer diseases mortality**						
Retinyl palmitate	0.9044 (0.7160–1.1425)	0.3995	0.5522 (0.3174–0.9606)	0.0355	0.4446 (0.2335–0.8466)	0.0136
Retinyl stearate	0.9609 (0.8760–1.0540)	0.3975	0.8277 (0.7159–0.9570)	0.0107	0.8156 (0.6803–0.9778)	0.0276

Model 1: No adjustment for covariates. Model 2: Adjusted for age, gender, and race. Model 3: Age, BMI, waist circumference, ALT, AST, race, education level, PIR, marital status, hypertension, diabetes, PreCVD, smoking status, and drinking status.

Among the examined biomarkers, vitamin D was significantly associated with reduced all-cause mortality [HR = 0.994, 95% CI: (0.9902–0.9978), *P* = 0.0022]. Similarly, higher levels of retinyl palmitate [0.7186 (0.5622–0.9186), *P* = 0.0083] and retinyl stearate [0.9024 (0.8479–0.9604), *P* = 0.0012] were also associated with decreased mortality risk. In the analysis of cardiovascular mortality, elevated vitamin C concentrations were significantly associated with increased risk of cardiovascular death [1.575 (1.0335–2.4002), *P* = 0.0346]. Regarding cancer-specific mortality, Model 3 demonstrated that higher levels of retinyl palmitate [0.4446 (0.2335–0.8466), *P* = 0.0136] and retinyl stearate [0.8156 (0.6803–0.9778), *P* = 0.0276] were significantly associated with a lower risk of death. No significant associations were observed between other serum vitamins or carotenoids and mortality risk in OA patients. Detailed results are provided in Supplementary Tables S2A–C.

### 3.3 Nonlinear association analysis

**Figure 3:**
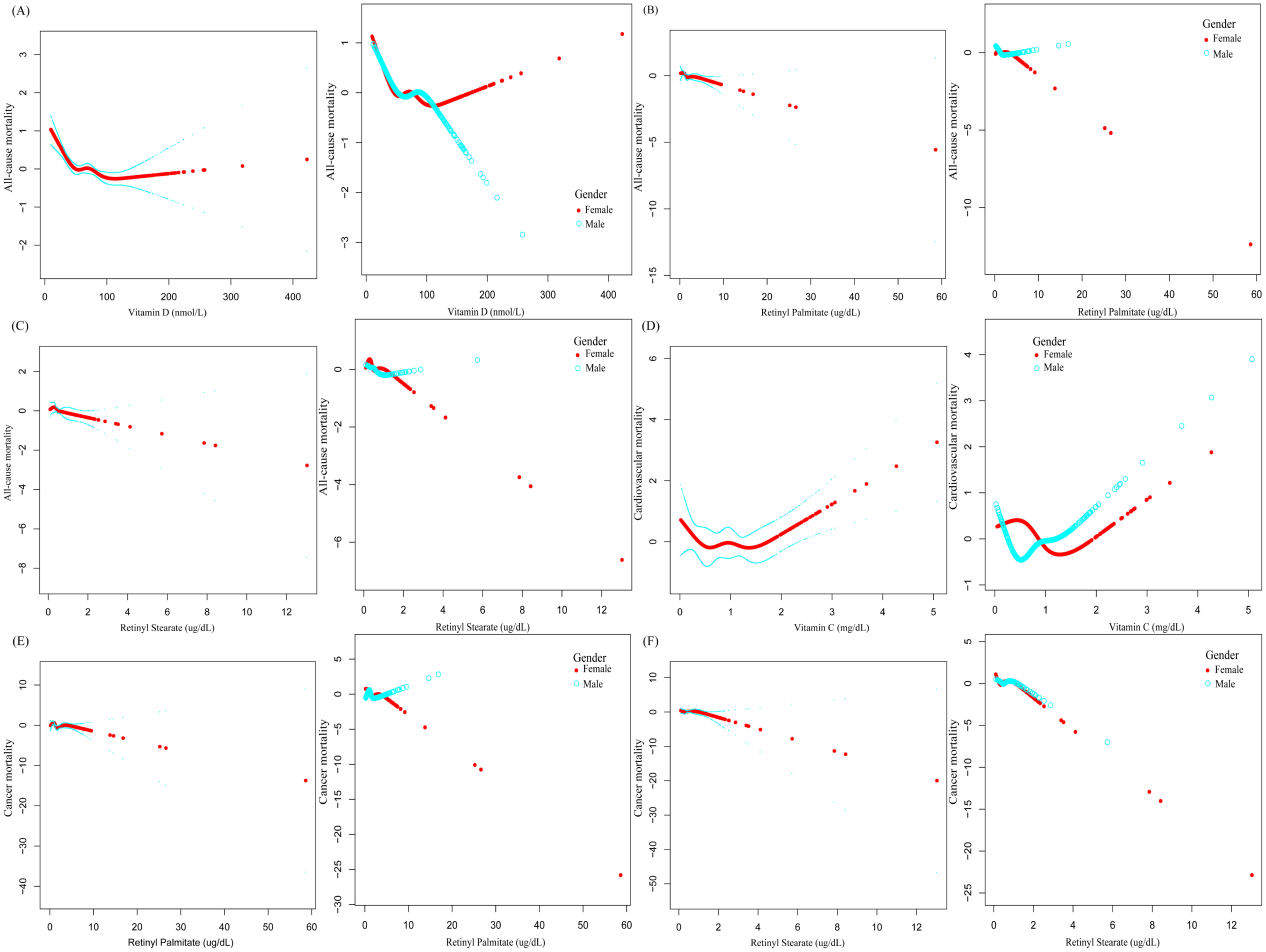
The association between serum vitamin, carotenoid, and retinyl ester levels and mortality risk in patients with OA.

Smoothing spline curves suggested potential nonlinear associations between serum vitamins, carotenoids, and retinyl esters levels and mortality risk among OA patients (Figure 3). To further examine threshold effects, two-piecewise Cox proportional hazards models were employed (Table 3). A significant inverse association was observed between serum vitamin D levels and all-cause mortality [0.99 (0.99–1.00), *P* < 0.0001]. The identified inflection point was 48.3 nmol/L. Below this threshold, each 1 nmol/L increase in serum vitamin D was associated with a 2% reduction in all-cause mortality risk [0.98 (0.97–0.99), *P* < 0.0001] above this level, the association was no longer statistically significant. A U-shaped association was detected between serum vitamin C levels and cardiovascular mortality. The inflection point was 1.15 mg/dl. Below this level, higher vitamin C concentrations were associated with decreased cardiovascular mortality (0.53, 0.23–1.25). However, above the threshold, higher levels were significantly associated with increased mortality risk [2.42 (1.45–4.04), *P* = 0.0007]. In subgroup analysis among female OA patients, serum vitamin D levels were inversely associated with all-cause mortality in a nonlinear pattern. The turning point was identified at 30.5 nmol/L. Below this level, each 1 nmol/L increase in vitamin D was associated with a 7% reduction in mortality risk [0.93 (0.90–0.96), *P* < 0.0001], whereas the association was not significant above this point (Supplementary Table S3). All log-likelihood ratio tests for nonlinear effects yielded *P* < 0.05, indicating statistically significant threshold effects.

**Table 3:** Threshold effect analysis of serum vitamins, carotenoids, and retinyl esters in relation to mortality risk among patients with OA.

**Mortality category**	**Adjusted HR (95% CI) *P*-value**
**All-cause mortality**	
**Vitamin D**	
Fitting by the standard linear model	0.99 (0.99–1.00) <0.0001
**Fitting by the two-piecewise linear model**	
Inflection point	48.3
<48.3 (nmol/L)	0.98 (0.97–0.99) <0.0001
>48.3 (nmol/L)	1.00 (0.99–1.00) 0.1512
Log likelihood ratio	<0.001
**Retinyl palmitate**	
Fitting by the standard linear model	0.91 (0.86–0.98) 0.0088
**Fitting by the two-piecewise linear model**	
Inflection point	1.7
<1.7 (μg/dl)	0.80 (0.60–1.05) 0.1046
>1.7 (μg/dl)	0.94 (0.87–1.02) 0.1189
Log likelihood ratio	0.312
**Retinyl stearate**	
Fitting by the standard linear model	0.77 (0.61–0.98) 0.0305
**Fitting by the two-piecewise linear model**	
Inflection point	1.38
<1.38 (μg/dl)	0.71 (0.48–1.05) 0.0844
>1.38 (μg/dl)	0.86 (0.55–1.33) 0.4861
Log likelihood ratio	0.604
**Cardiovascular disease mortality**	
**Vitamin C**	
Fitting by the standard linear model	1.40 (0.86–2.30) 0.1787
**Fitting by the two-piecewise linear model**	
Inflection point	1.15
<1.15 (mg/dl)	0.53 (0.23–1.25) 0.1454
>1.15 (mg/dl)	2.42 (1.45–4.04) 0.0007
Log likelihood ratio	0.011
**Cancer diseases mortality**	
**Retinyl palmitate**	
Fitting by the standard linear model	0.84 (0.70–1.01) 0.0699
**Fitting by the two-piecewise linear model**	
Inflection point	1.6
<1.6 (μg/dl)	0.51 (0.27–0.97) 0.0393
>1.6 (μg/dl)	0.94 (0.77–1.14) 0.5346
Log likelihood ratio	0.112
**Retinyl stearate**	
Fitting by the standard linear model	0.47 (0.23–0.95) 0.0361
**Fitting by the two-piecewise linear model**	
Inflection point	1.3
<1.3 (μg/dl)	0.76 (0.29–2.00) 0.5841
>1.3 (μg/dl)	0.03 (0.00–7.37) 0.2116
Log likelihood ratio	0.146

Adjusted for age, BMI, waist circumference, ALT, AST, race, education level, PIR, marital status, hypertension, diabetes, PreCVD, smoking status, and drinking status.

### 3.4 Subgroup analysis

Subgroup analyses revealed that the inverse association between serum vitamin D levels and all-cause mortality in OA patients was more pronounced among individuals with lower educational attainment. Specifically, this association remained statistically significant in participants with <9th grade education (HR = 0.99, *P* = 0.0045) and those with a high school diploma or equivalent (HR = 0.99, *P* = 0.0018), but was not significant in individuals with higher education levels (Table 4). Additionally, in participants with <9th grade education, higher levels of serum retinyl stearate were significantly associated with an increased risk of all-cause mortality [1.45 (1.09–1.92), *P* = 0.0107], whereas no such association was observed among those with higher education (Supplementary Table S4). The *P*-values for interaction were <0.05 for both analyses, indicating significant effect modification by education level. No significant effect modification was observed in other predefined subgroups (detailed in Supplementary Table S4).

**Table 4:** Subgroup analysis of the association between serum vitamin D and all-cause mortality.

**Subgroup**	**Adjusted HR (95% CI) *P***	***P* for interaction**
**Drinking status**		0.9996
Never	0.99 (0.99–1.00) 0.0456	
Former	0.99 (0.99–1.00) 0.0150	
Mild	0.99 (0.99–1.00) 0.0021	
Moderate	1.00 (0.99–1.01) 0.3676	
Severe	1.00 (0.99–1.00) 0.3252	
**Hypertension**		
No	0.99 (0.99–1.00) 0.0012	
Yes	0.99 (0.99–1.00) 0.0008	
**PIR**		0.6361
Low	1.00 (0.99–1.00) 0.1463	
Middle	0.99 (0.99–1.00) 0.0154	
High	0.99 (0.98–1.00) 0.0011	
**Gender**		0.2781
Female	0.99 (0.99–1.00) 0.0012	
Male	0.99 (0.99–1.00) 0.0026	
**Race**		0.4546
Other Race—Including Multi-Racial	0.98 (0.97–1.00) 0.0108	
Mexican American	0.99 (0.98–1.00) 0.1383	
Other Hispanic	0.99 (0.97–1.00) 0.1387	
Non-Hispanic White	0.99 (0.99–1.00) <0.0001	
Non-Hispanic Black	0.99 (0.98–1.00) 0.0072	
**Education level**		0.0425
College graduate or above	1.00 (0.99–1.00) 0.4188	
Less than 9th grade	0.99 (0.98–1.00) 0.0045	
9–11th grade (Includes 12th grade with no diploma)	1.00 (0.99–1.00) 0.4121	
High school graduate/GED or equivalent	0.99 (0.98–1.00) 0.0018	
Some college or AA degree	0.99 (0.99–1.00) 0.0527	
**Marital status**		0.9519
Never married	1.00 (0.99–1.01) 0.7984	
Married	0.99 (0.99–1.00) 0.0036	
Widowed	0.99 (0.99–1.00) 0.0052	
Divorced	1.00 (0.99–1.00) 0.1862	
Separated	1.01 (0.98–1.03) 0.5654	
Living with partner	1.00 (0.99–1.02) 0.7606	
**Smoking status**		0.4258
Never	0.99 (0.99–1.00) 0.0104	
Former	1.00 (0.99–1.00) 0.0248	
Now	0.99 (0.99–1.00) 0.0193	
**PreCVD**		0.8135
No	0.99 (0.99–1.00) 0.0018	
Yes	0.99 (0.99–1.00) 0.0025	
**Diabetes**		0.7237
No	0.99 (0.99–1.00) <0.0001	
Yes	1.00 (0.99–1.00) 0.0595	

All adjustment variables except the variables themselves were adjusted as above.

### 3.5 Sensitivity analysis

In the sensitivity analysis excluding participants who died within the first two years of follow-up, the fully adjusted Model 3 for all-cause mortality showed consistent inverse associations: vitamin D [0.9938 (0.9888–0.9989), *P* = 0.0162], retinyl palmitate [0.8866 (0.8281–0.9493), *P* = 0.0005], and retinyl stearate [0.6744 (0.4995–0.9105), *P* = 0.0101]. For cardiovascular mortality, the association with vitamin C was not statistically significant [1.8393 (0.7651–4.4219), *P* = 0.1733]. For cancer-related mortality, retinyl palmitate [0.852 (0.6871–1.0566), *P* = 0.1446] and retinyl stearate [0.5152 (0.2495–1.0638), *P* = 0.0730] showed similar trends as in the main analysis, though statistical significance was not reached. In the second sensitivity analysis excluding participants with pre-existing cardiovascular disease (Pre-CVD), the inverse association between serum vitamin D and all-cause mortality remained significant [0.9949 (0.9901–0.9997), *P* = 0.0374], as did the association with retinyl palmitate [0.911 (0.8326–0.9968), *P* = 0.0424]. The association between retinyl stearate and all-cause mortality was attenuated and no longer statistically significant [0.7607 (0.5084–1.1382), *P* = 0.1834]. For cardiovascular mortality, vitamin C again showed a non-significant positive association [1.7054 (0.8961–3.2455), *P* = 0.1040]. For cancer mortality, both retinyl palmitate [0.7851 (0.5817–1.0596), *P* = 0.1138] and retinyl stearate [0.5603 (0.2612–1.2015), *P* = 0.1367] remained non-significant but directionally consistent with the main findings. Baseline and sensitivity analysis results for all-cause, cardiovascular, and cancer-specific mortality are presented in Supplementary Tables S5, S6, 7A, B.

### 3.6 Associations between serum vitamins, carotenoids, retinyl esters, and mortality risk in non-OA participants

To assess whether the associations identified in Section 3.2 were specific to individuals with OA, we conducted parallel analyses in participants without OA. In the analysis of all-cause mortality using Model 3, serum vitamin D remained significantly associated with lower mortality risk [0.994 (0.9923–0.9957), *P* < 0.0001], while no significant associations were observed for retinyl palmitate [1.0084 (0.9903–1.0267), *P* = 0.3662] or retinyl stearate [1.0414 (0.9939–1.0912), *P* = 0.0888].

For cardiovascular mortality, vitamin C levels were not significantly associated with mortality risk [0.8834 (0.7429–1.0504), *P* = 0.1605]. Regarding cancer mortality, neither retinyl palmitate [1.018 (0.9846–1.0525), *P* = 0.2948) nor retinyl stearate [1.0376 (0.9232–1.1663), *P* = 0.5355) was associated with mortality risk. These findings suggest that the observed associations in OA patients may not extend to the general population. Full results are presented in Supplementary Table S8.

### 3.7 Combined effects of serum vitamins, carotenoids, retinyl esters levels and OA status on mortality risk

To further examine the interaction between serum nutrient levels and OA status, participants were stratified into four groups based on OA status (yes/no) and serum vitamin or carotenoid levels (low/high, dichotomized at the median). In Model 3 for all-cause mortality: participants with low vitamin D and OA [Low/OA (+)] had significantly increased mortality risk compared to the reference group [Low/OA (–)] [1.1959 (1.0297–1.3890), *P* = 0.0191]. In contrast, those without OA and with high vitamin D levels [High/OA (–)] had significantly reduced risk [0.7808 (0.7255–0.8402), *P* < 0.0001]. For retinyl palmitate, the [Low/OA (+)] group also had higher mortality risk [1.3914 (1.1195–1.7294), *P* = 0.0029], while the [High/OA (+)] group showed no significant difference. Similarly, for retinyl stearate, the [Low/OA (+)] group showed elevated mortality risk [1.4084 (1.0946–1.8122), *P* = 0.0077], with no significant association in the [High/OA (+)] group. These results suggest that higher levels of vitamin D, retinyl palmitate, and retinyl stearate may attenuate the detrimental effect of OA on all-cause mortality risk. In the analysis of cancer-specific mortality, participants with low retinyl palmitate levels and OA [Low/OA (+)] had a significantly increased risk compared to [Low/OA (–)] [1.6143 (1.0608–2.4564), *P* = 0.0254], while no significant association was observed in the high-level group. These findings further support a potential protective effect of higher retinyl palmitate levels in individuals with OA (Table 5).

**Table 5:** Association between serum vitamins, carotenoids, retinyl esters, and OA status in relation to mortality.

**Group**	**Model 1**		**Model 2**		**Model 3**	
	**HR (95% CI)**	***P*** **-value**	**HR (95% CI)**	***P*** **-value**	**HR (95% CI)**	***P*** **-value**
**All-cause mortality**						
**Vitamin D**						
Low /OA–	ref		ref		ref	
Low /OA+	2.4472 (2.0876–2.8686)	<0.0001	1.3072 (1.1302–1.5120)	0.0003	1.1959 (1.0297–1.3890)	0.0191
High/OA–	0.8451 (0.7806–0.9148)	<0.0001	0.6861 (0.6384–0.7374)	<0.0001	0.7808 (0.7255–0.8402)	<0.0001
High/OA+	2.2346 (1.9069–2.6186)	<0.0001	0.8863 (0.7672–1.0238)	0.1009	0.9289 (0.8045–1.0725)	0.3144
**Retinyl palmitate**						
Low /OA–	ref		ref		ref	
Low /OA+	2.6676 (2.0708–3.4363)	<0.0001	1.5979 (1.2894–1.9801)	<0.0001	1.3914 (1.1195–1.7294)	0.0029
High/OA–	1.0442 (0.9511–1.1463)	0.3643	0.862 (0.7851–0.9464)	0.0018	1.0112 (0.9240–1.1065)	0.8092
High/OA+	2.7849 (2.1647–3.5827)	<0.0001	1.1221 (0.9154–1.3755)	0.2674	1.048 (0.8527–1.2881)	0.6558
**Retinyl stearate**						
Low /OA–	ref		ref		ref	
Low /OA+	2.9667 (2.2740–3.8704)	<0.0001	1.6667 (1.3128–2.1161)	<0.0001	1.4084 (1.0946–1.8122)	0.0077
High/OA–	1.3563 (1.2036–1.5283)	<0.0001	0.9158 (0.8311–1.0092)	0.0759	0.981 (0.8929–1.0779)	0.6904
High/OA+	3.1638 (2.4333–4.1137)	<0.0001	1.1412 (0.9002–1.4465)	0.2751	0.9941 (0.7860–1.2573)	0.9606
**Cardiovascular disease mortality**						
**Vitamin C**						
Low /OA–	ref		ref		ref	
Low /OA+	2.5744 (1.3853–4.7840)	0.0028	1.2919 (0.6937–2.4059)	0.4195	1.0558 (0.5544–2.0106)	0.8688
High/OA–	0.9379 (0.7652–1.1495)	0.5367	1.6555 (1.3277–2.0641)	<0.0001	1.1681 (0.9461–1.4421)	0.1486
High/OA+	1.9897 (1.2205–3.2438)	0.0058	1.7061 (1.1744–2.4787)	0.0051	1.0201 (0.6837–1.5219)	0.9225
**Cancer diseases mortality**						
**Retinyl palmitate**						
Low /OA–	ref		ref		ref	
Low /OA+	3.0116 (1.9310–4.6968)	<0.0001	1.8524 (1.1901–2.8835)	0.0063	1.6143 (1.0608–2.4564)	0.0254
High/OA–	1.0301 (0.8550–1.2409)	0.7553	0.8708 (0.7270–1.0431)	0.1331	1.021 (0.8489–1.2280)	0.8252
High/OA+	1.7804 (0.9855–3.2164)	0.0559	0.7775 (0.4284–1.4111)	0.4080	0.7588 (0.4173–1.3798)	0.3657
**Retinyl stearate**						
Low /OA–	ref		ref		ref	
Low /OA+	2.5403 (1.5507–4.1614)	0.0002	1.4677 (0.8739–2.4650)	0.1469	1.2143 (0.7135–2.0666)	0.4743
High/OA–	1.1759 (0.9348–1.4793)	0.1664	0.8248 (0.6658–1.0218)	0.0779	0.8606 (0.6840–1.0828)	0.2002
High/OA+	2.5154 (1.3703–4.6172)	0.0029	0.9913 (0.5251–1.8713)	0.9784	0.903 (0.4896–1.6656)	0.7440

## 4 Discussion

To our knowledge, this is the first nationwide, population-based study to systematically assess the associations between multiple serum vitamins, carotenoids, retinyl esters, and mortality risk among individuals with OA. This study yields three principal findings. First, elevated serum levels of vitamin D, retinyl palmitate, and retinyl stearate were significantly associated with reduced risk of all-cause mortality in OA patients. These associations remained robust across sensitivity analyses. Second, a U-shaped association was observed between serum vitamin C and cardiovascular mortality, suggesting the existence of a critical threshold in its usage and effect. Third, higher levels of retinyl palmitate and retinyl stearate were also associated with reduced cancer-specific mortality, highlighting their potential role in cancer prognosis among OA patients.

Moreover, nonlinear modeling revealed a significant threshold effect between vitamin D levels and all-cause mortality risk, with a turning point at 48.3 nmol/L. Below this threshold, vitamin D appeared protective, but no further benefit was observed beyond this level. This aligns with findings by Xu et al. (17), who reported that serum vitamin D levels exceeding 84.8 nmol/L were no longer associated with bone mineral density (BMD), with a notably lower threshold in females. Interestingly, our study similarly identified a much lower turning point for vitamin D in female OA patients—only 30.5 nmol/L. This suggests that women with OA may be more vulnerable to even mild vitamin D deficiency, and that subclinical insufficiency could elevate their mortality risk. As such, clinical strategies for vitamin D supplementation in this population should focus not only on overt deficiency but also on avoiding “borderline insufficiency.” Consistent patterns have been reported in studies of older adults with heart failure, where women were more likely to fall into low vitamin D categories and had markedly higher all-cause mortality (up to 26% in the lowest tertile group) (18). Vitamin D deficiency is also widely associated with adverse outcomes in chronic conditions such as cardiovascular disease, chronic kidney disease, and osteoporosis (19–23), underscoring its clinical importance. Notably, although higher levels of vitamin D did not appear to confer additional survival benefit in our study, “L-shaped” or even “U-shaped” relationships have been previously reported in other populations (24, 25). Our findings regarding the U-shaped association of vitamin C with cardiovascular mortality also suggest a potential threshold-dependent effect. However, a recent umbrella review analyzing multiple RCTs found that high-dose intravenous vitamin C (≥6 g/day), while occasionally associated with side effects such as hypernatremia and oxalate nephropathy, did not significantly alter the risk of major cardiovascular events compared to placebo (26). Most evidence to date suggests that normal or moderately high levels of vitamin C exert antioxidant and vasoprotective effects, particularly in populations with deficiency or high cardiovascular risk, rather than among the general healthy population (27). Nonetheless, the role of vitamin C in cardiovascular mortality remains controversial, and the U-shaped relationship observed here should be interpreted cautiously (28, 29).

To explore potential effect modifications across different strata, we conducted further subgroup analyses. The results showed that the protective effects of serum vitamin D and retinyl stearate on all-cause mortality in OA patients were more pronounced among individuals with lower educational attainment, particularly those with less than a 9th-grade education. These findings are consistent with previous studies. For example, Wang et al. (30) reported that individuals with higher vitamin D levels tended to have favorable characteristics such as higher educational attainment, nonsmoking status, and lower BMI, all of which are independently associated with reduced mortality risk. Interestingly, although educational level showed significant heterogeneity in our subgroup analysis, we did not observe similar differences across strata defined by the PIR, a common measure of economic status. This suggests that for nutrients such as vitamins—relatively easy to access—the strength of their association with mortality risk may not be primarily determined by economic conditions *per se*, but rather by health literacy shaped by educational background. Stormacq et al. (31) identified education as the most critical determinant of health literacy, which in turn mediates the relationship between socioeconomic status and health outcomes. In other words, it may not be that people “can't afford to eat well,” but rather that they “don't know how to eat well,” underscoring the potential public health value of promoting health education in low-education populations (32).

Vitamin D, vitamin C, as well as retinyl palmitate and retinyl stearate, are all essential micronutrients for the human body. Vitamin D is a fat-soluble vitamin that primarily exists in two forms: D2 (ergocalciferol) and D3 (cholecalciferol) (33). Its main functions include promoting the absorption of calcium and phosphorus, maintaining stable blood calcium levels, and facilitating bone mineralization. Additionally, it plays a crucial role in immune system regulation (34, 35). Vitamin D can be synthesized in the skin through exposure to ultraviolet (UV) rays, or obtained through the consumption of vitamin D-rich foods such as fatty fish, egg yolks, and liver, as well as fortified foods like vitamin D-fortified milk (36). Vitamin C (ascorbic acid) is a water-soluble vitamin with strong antioxidant properties. It scavenges free radicals in the body, reduces oxidative stress, and promotes collagen synthesis, thereby benefiting the health of bones, skin, and blood vessels (29, 37). In addition, vitamin C is involved in iron absorption and the maintenance of immune function (38). As the human body cannot synthesize vitamin C on its own, it mainly relies on dietary intake from fresh fruits (such as citrus fruits and strawberries) and vegetables (such as broccoli and spinach) (39). Retinyl Palmitate and Retinyl Stearate are esterified storage forms of vitamin A formed by the combination of retinol and fatty acids. They are mainly found in animal liver, whole-fat dairy products, and eggs (40). After ingestion, they can be hydrolyzed in the body into the active form of retinol, which participates in physiological processes such as retinal function, maintenance of skin and mucosal barriers, and immune responses (41–43).

It is worth noting that although vitamin A has been reported to benefit bone development in adolescents (44, 45), excessive intake of vitamin A—particularly through high-dose supplements—has been confirmed to be associated with neurotoxicity, hepatotoxicity, decreased bone mineral density, and an increased risk of fractures in the elderly population (46–49). Moreover, current toxicity studies on retinyl palmitate have primarily focused on its phototoxicity and the potential for inducing mood-related changes with long-term use (50–52). Research on whether retinyl palmitate or stearate themselves have potential toxic effects at appropriate doses remains limited, and no definitive conclusions have been reached. Overall, these micronutrients enjoy wide public acceptance and accessibility. Compared with other interventions, nutritional approaches are more suitable for promotion within primary healthcare systems, particularly in resource-limited settings. If well-implemented, targeted nutritional supplementation programs at the primary care level hold promise as effective strategies to improve the health of patients with OA and help reduce healthcare disparities. However, large-scale prospective studies across diverse populations and regions are still needed to validate their effectiveness and optimize application strategies.

In addition, because our primary analyses focused on individuals with OA, we conducted verification analyses among non-OA participants to assess whether the observed associations were specific to the OA population. In the non-OA group, no significant associations were observed between retinyl palmitate, retinyl stearate, and mortality outcomes. This suggests that the protective effects of these compounds on mortality may be specific to individuals with OA, possibly due to disease-specific pathological mechanisms. Currently, research on the roles of retinyl palmitate and retinyl stearate in OA remains scarce. Previous studies have only reported that retinyl palmitate may modulate cytogenotoxic events induced by cyclophosphamide (CPA) and doxorubicin (DOX) (53). Barker et al. (54) found no evidence that retinyl palmitate adversely affects fracture risk. Additionally, low levels of retinyl palmitate and retinyl stearate have been linked to peripheral artery disease and cognitive impairment in older adults (55, 56). Although the precise mechanisms remain unclear, it is plausible that these compounds may exert their protective effects in OA by mitigating inflammation, suppressing local oxidative stress in the joints, or modulating related signaling pathways. Given the paucity of research in this area, further investigations are warranted to elucidate their potential mechanistic roles and therapeutic value in OA-related outcomes.

In the final validation analysis combining serum vitamins, carotenoids, retinyl esters levels and OA status, we found that among participants with low levels of serum vitamin D, retinyl palmitate, or retinyl stearate, those with OA had significantly higher mortality risk compared to the non-OA reference group. This highlights the adverse impact of OA on survival. However, in the High/OA (+) groups, no significant difference in mortality risk was observed compared to the reference group. Notably, the High/OA (+) group for vitamin D even showed a significantly lower risk of death. These findings suggest that higher serum levels of vitamin D, retinyl palmitate, and retinyl stearate may effectively offset the detrimental effect of OA on mortality, further supporting their potential role in improving survival among OA patients. Future large-scale clinical studies are warranted to validate these findings and to explore the feasibility of these nutrients as targeted interventions in OA management.

In this study, serum data for B vitamins, folate, and vitamin K were largely missing across different NHANES cycles. Therefore, these variables were not included in the analysis. Given the potential roles of these nutrients in the pathogenesis of OA and in chronic disease management, future research should investigate these associations when complete data become available. Additionally, this study did not adjust for dietary intake or the use of vitamin supplements, which may have influenced the accuracy of serum nutrient levels and introduced potential confounding bias. Although serum concentrations can serve as an integrated marker of nutritional status, they may not fully reflect long-term exposure and are susceptible to recent dietary or supplement-related fluctuations. Thus, future studies should incorporate comprehensive dietary assessments and detailed information on supplement use to validate and expand upon our findings. Moreover, OA status in the NHANES database is based on self-reported physician diagnoses, without information on disease duration, severity, or progression. This limitation restricts our ability to assess the long-term role of serum nutrients in OA management. Therefore, the findings of this study should be interpreted with caution, and prospective cohort studies with complete clinical documentation are warranted to further explore these associations. Although the number of cancer-related deaths in this study was sufficient to support the primary analysis, statistical power may still be limited for more granular subgroup analyses, such as those focusing on specific cancer types or populations. Lastly, the identification of OA in NHANES relied on participants' responses to the question, “Has a doctor ever told you that you had osteoarthritis?” While this approach is widely used in large-scale epidemiological studies and administered by trained personnel via the CAPI system to minimize systematic bias, the risk of recall bias and misclassification remains, especially in the absence of radiographic or clinical verification. Future research should incorporate objective diagnostic criteria to enhance the accuracy of OA classification and the reliability of study outcomes.

This study has several notable strengths: (1) The use of nationally representative data from a large population-based sample enhances the generalizability of our findings. (2) To our knowledge, this is the first study to comprehensively evaluate the associations between multiple serum vitamins, carotenoids, retinyl esters, and mortality in OA patients. (3) A robust, multi-level analytic approach—including nonlinear modeling, sensitivity analyses, subgroup analyses, and replication in non-OA controls—was employed to enhance the credibility and applicability of our conclusions. Nonetheless, certain limitations should be acknowledged. First, we cannot completely rule out the possibility of residual confounding. One notable limitation of this study is the absence of urban-rural residency status as a potential confounder. Although we partially accounted for socioeconomic status using the PIR, the NHANES dataset does not explicitly classify participants based on urban or rural residence. However, substantial differences may exist between urban and rural populations in terms of dietary patterns, food sources, and lifestyle behaviors. For instance, individuals living in rural areas may consume more natural, unprocessed, or self-produced foods, whereas urban residents may have greater access to processed foods and dietary supplements. Differences in dietary and lifestyle factors may lead to variations in serum nutrient levels, potentially influencing the associations observed with bone health outcomes. The lack of this variable may introduce a degree of confounding bias, warranting further consideration and validation in future studies. Moreover, this study did not incorporate genetic factors as potential confounders. Individual genetic backgrounds may influence not only susceptibility to OA but also the absorption, metabolism, and distribution of vitamins in the body, which could in turn affect the associations between serum vitamin levels and OA or mortality observed in this study. Due to the lack of comprehensive genotyping data in the NHANES survey cycles utilized, we were unable to account for residual confounding attributable to genetic variation. Future research should aim to integrate genetic information to better elucidate the potential causal relationships between vitamin levels and the development and prognosis of OA.

## 5 Conclusion

Among individuals with OA, higher serum levels of vitamin D, retinyl palmitate, and retinyl stearate were associated with a lower risk of mortality, with the associations being particularly evident in women and individuals with lower educational attainment. These findings provide important epidemiological evidence to support individualized nutritional intervention strategies in OA management. Further large-scale clinical studies are warranted to validate these associations and elucidate the underlying biological mechanisms.

## Data Availability

Publicly available datasets were analyzed in this study. The raw data and analysis code supporting the findings of this study are not publicly available but can be obtained from the corresponding author upon reasonable request. Currently, the data are not deposited in any public repository and therefore do not have an accession number.

## Supplementary material

Supplementary Figure 1A Flow chart (vitamin C).

Supplementary Figure 1B Flow chart (vitamin E, A, carotenoids, vitamin A esters).

Supplementary Table S1 Baseline characteristics of the OA patient population based on sex and vitamin C and D.

Supplementary Table S2A Cox regression analysis of serum vitamins, carotenoids, and retinyl esters and their non-significant associations with all-cause mortality in OA patients.

Supplementary Table S2B Cox regression analysis of serum vitamins, carotenoids, and retinyl esters and their non-significant associations with cardiovascular mortality in OA patients.

Supplementary Table S2C Cox regression analysis of serum vitamins, carotenoids, and retinyl esters and their non-significant associations with cancer mortality in OA patients.

Supplementary Table S3 Gender-stratified threshold effect analysis of serum vitamins, carotenoids, and retinyl esters and mortality risk in OA patients.

Supplementary Table S4 Subgroup analyses in other sections.

Supplementary Table S5 Baseline characteristics of participants who died within the first 2 years of follow-up.

Supplementary Table S6 Baseline characteristics excluding participants with preCVD.

Supplementary Table S7A Cox regression analysis of serum vitamins, carotenoids, and retinyl esters and mortality in OA patients, excluding early deaths.

Supplementary Table S7B Cox regression analysis of serum vitamins, carotenoids, and retinyl esters and mortality in OA patients, excluding participants with pre-existing CVD.

Supplementary Table S8 Cox regression analysis of serum vitamins, carotenoids, and retinyl esters and mortality risk in participants without OA.
