# Supplementary material for: Do serum vitamins, carotenoids, and retinyl esters influence mortality in osteoarthritis? Insights from a nationally representative study
Source: Front Nutr. 2025 Jun 19;12:1609759. doi: 10.3389/fnut.2025.1609759 (PMC12224656; doi:10.3389/fnut.2025.1609759)
Supplement: Supplementary Figure 1A — Flow chart (vitamin C). [file Data_Sheet_1.zip › Data Sheet 1 (2)/Supplementary Material 1.docx]

Definitions of Clinical Conditions

1. Hypertension was defined as meeting any of the following criteria: (1) self-reported physician diagnosis of hypertension; (2) current use of antihypertensive medications; or (3) an average systolic blood pressure (SBP) ≥140 mmHg and/or diastolic blood pressure (DBP) ≥90 mmHg based on three standardized measurements.

2. Diabetes mellitus was defined as meeting one or more of the following: (1) self-reported physician diagnosis of diabetes; (2) glycated hemoglobin (HbA1c) level ≥6.5%; (3) fasting plasma glucose (FPG) level ≥7.0 mmol/L; or (4) current use of antidiabetic medications.

3. Cardiovascular disease (CVD) was identified based on self-reported physician diagnosis using a standardized medical questionnaire. Participants were classified as having CVD if they reported a history of coronary heart disease, congestive heart failure, myocardial infarction, angina pectoris, or stroke.

4. Smoking status was categorized into three groups: (1) “Never” (smoked fewer than 100 cigarettes in their lifetime); (2) “Former” (smoked at least 100 cigarettes but had quit); (3) “Now” (current smokers).

5. Alcohol consumption was classified into five categories: (1) “Never” (consumed <12 alcoholic drinks in their lifetime); (2) “Former” (consumed ≥12 alcoholic drinks per year previously but had abstained in the past year); (3) “Mild” (≤1 drink/day for women, ≤2 drinks/day for men); (4) “Moderate” (2 drinks/day for women, 3 drinks/day for men); (5) “Severe” (>2 drinks/day for women, >3 drinks/day for men).

Laboratory Methods for the Measurement of Serum Vitamins

1.Vitamin A and its related compounds, including carotenoids (α-carotene, trans-β-carotene, cis-β-carotene, β-cryptoxanthin, lutein/zeaxanthin, and trans-lycopene) and retinyl esters (retinyl palmitate and retinyl stearate), were measured using high-performance liquid chromatography (HPLC) with a photodiode array detector, and concentrations were expressed in µg/dL.

2.Vitamin C was measured using ultra-performance liquid chromatography with electrochemical detection (UPLC-ECD), and results were reported in mg/dL.

3.Vitamin D concentrations were determined by high-performance liquid chromatography coupled with tandem mass spectrometry (HPLC-MS/MS) and expressed in nmol/L. Vitamin E was quantified using HPLC with photodiode array detection and reported in µg/dL.
