# Supplementary material for: Do serum vitamins, carotenoids, and retinyl esters influence mortality in osteoarthritis? Insights from a nationally representative study
Source: Front Nutr. 2025 Jun 19;12:1609759. doi: 10.3389/fnut.2025.1609759 (PMC12224656; doi:10.3389/fnut.2025.1609759)
Supplement: Supplementary Figure 1A — Flow chart (vitamin C). [file Data_Sheet_1.zip › Data Sheet 1 (2)/Supplementary Table 1.docx]

Supplementary Table S1 Baseline characteristics of the OA patient population based on sex and vitamin C and D

| Variables | Female | Male | *P*-value |
| --- | --- | --- | --- |
| *For continuous variables, mean (95% CI)* | | | |
| Age (years) | 61.49 (60.05–62.93) | 59.98 (58.47–61.50) | 0.1723 |
| BMI (kg/m²) | 31.10 (29.82–32.37) | 30.27 (29.49–31.05) | 0.3427 |
| Waist circumference (cm) | 101.90 (99.32–104.47) | 107.62 (105.87–109.38) | 0.0011 |
| ALT (U/L) | 20.00 (18.50–21.51) | 25.54 (22.81–28.26) | 0.0008 |
| AST (U/L) | 20.00 (18.50–21.51) | 25.54 (22.81–28.26) | 0.0008 |
| Vitamin C (mg/dL) | 1.04 (0.97–1.10) | 0.86 (0.79–0.92) | <0.0001 |
| *For categorical variables, percentage (95% CI)* | | | |
| Race |  |  | 0.4262 |
| Other Race - Including Multi-Racial | 6.64 (4.19–10.37) | 8.24 (4.62–14.27) |  |
| Mexican American | 4.67 (3.33–6.52) | 3.14 (1.84–5.28) |  |
| Other Hispanic | 3.41 (2.22–5.20) | 2.01 (0.77–5.10) |  |
| Non-Hispanic White | 74.34 (69.09–78.96) | 78.31 (71.27–84.02) |  |
| Non-Hispanic Black | 10.94 (8.27–14.32) | 8.31 (5.93–11.53) |  |
| Education level |  |  | 0.0254 |
| College graduate or above | 20.36 (16.11–25.40) | 30.34 (24.02–37.49) |  |
| Less than 9th grade | 4.20 (2.92–6.00) | 4.70 (3.07–7.13) |  |
| 9-11th grade (Includes 12th grade with no diploma) | 8.57 (6.71–10.88) | 10.28 (7.51–13.93) |  |
| High school graduate/GED or equivalent | 31.45 (26.34–37.05) | 26.49 (21.86–31.71) |  |
| Some college or AA degree | 35.42 (28.98–42.44) | 28.19 (22.88–34.18) |  |
| PIR |  |  | 0.1804 |
| Low | 22.46 (18.26–27.32) | 16.69 (12.23–22.36) |  |
| Middle | 39.01 (33.43–44.89) | 38.87 (33.15–44.91) |  |
| High | 38.53 (32.26–45.20) | 44.44 (37.00–52.14) |  |
| Marital status |  |  | 0.0004 |
| Never married | 7.52 (4.41–12.53) | 6.16 (3.19–11.55) |  |
| Married | 51.78 (46.08–57.43) | 67.82 (60.49–74.37) |  |
| Widowed | 20.98 (16.03–26.98) | 6.72 (4.20–10.59) |  |
| Divorced | 13.69 (10.01–18.44) | 13.10 (8.52–19.62) |  |
| Separated | 2.06 (1.30–3.24) | 1.37 (0.66–2.83) |  |
| Living with partner | 3.97 (2.44–6.41) | 4.82 (2.72–8.42) |  |
| Hypertension |  |  | 0.5003 |
| No | 45.58 (40.16–51.11) | 42.66 (34.94–50.76) |  |
| Yes | 54.42 (48.89–59.84) | 57.34 (49.24–65.06) |  |
| Diabetes |  |  | 0.7159 |
| No | 76.57 (71.39–81.06) | 77.57 (72.68–81.81) |  |
| Yes | 23.43 (18.94–28.61) | 22.43 (18.19–27.32) |  |
| PreCVD |  |  | 0.9110 |
| No | 77.10 (71.34–81.99) | 76.76 (71.35–81.41) |  |
| Yes | 22.90 (18.01–28.66) | 23.24 (18.59–28.65) |  |
| Smoking status |  |  | <0.0001 |
| Never | 54.71 (48.16–61.11) | 28.68 (22.56–35.70) |  |
| Former | 27.13 (22.75–32.00) | 47.46 (40.93–54.07) |  |
| Now | 18.16 (14.15–22.99) | 23.87 (19.05–29.46) |  |
| Drinking status |  |  | <0.0001 |
| Never | 13.35 (9.89–17.77) | 4.85 (2.76–8.36) |  |
| Former | 20.82 (17.75–24.26) | 20.85 (15.01–28.21) |  |
| Mild | 37.43 (32.47–42.67) | 39.34 (33.21–45.83) |  |
| Moderate | 18.32 (14.84–22.40) | 11.54 (6.88–18.71) |  |
| Severe | 10.08 (6.37–15.60) | 23.43 (17.08–31.24) |  |

For continuous variables: survey-weighted mean (95% CI), P-value was by survey-weighted linear regression

For categorical variables: survey-weighted percentage (95% CI), P-value was by survey-weighted Chi-square test

Table S1B Baseline characteristics of the OA patient population based on gender and vitamin D

| Variables | Female | Male | *P*-value |
| --- | --- | --- | --- |
| *For continuous variables, mean (95% CI)* | | | |
| Age (years) | 61.50 (60.77–62.22) | 59.68 (58.81–60.56) | 0.0032 |
| BMI (kg/m²) | 31.21 (30.65–31.76) | 30.48 (30.06–30.89) | 0.0421 |
| Waist circumference (cm) | 102.28 (101.20–103.35) | 107.79 (106.79–108.79) | <0.0001 |
| ALT (U/L) | 21.53 (20.79–22.28) | 27.52 (26.33–28.71) | <0.0001 |
| AST (U/L) | 24.12 (23.26–24.98) | 26.44 (25.47–27.40) | 0.0004 |
| Vitamin D (nmol/L) | 80.46 (78.10–82.81) | 74.30 (72.27–76.32) | <0.0001 |
| *For categorical variables, percentage (95% CI)* | | | |
| Race |  |  | 0.1057 |
| Other Race - Including Multi-Racial | 5.26 (4.15–6.63) | 6.40 (4.64–8.75) |  |
| Mexican American | 3.87 (3.08–4.85) | 3.59 (2.64–4.85) |  |
| Other Hispanic | 3.32 (2.62–4.20) | 2.80 (1.96–3.98) |  |
| Non-Hispanic White | 77.61 (75.11–79.92) | 79.54 (76.26–82.47) |  |
| Non-Hispanic Black | 9.95 (8.47–11.65) | 7.68 (6.32–9.30) |  |
| Education level |  |  | <0.0001 |
| College graduate or above | 23.50 (20.89–26.33) | 33.08 (29.15–37.27) |  |
| Less than 9th grade | 5.19 (4.24–6.34) | 5.21 (4.18–6.49) |  |
| 9-11th grade (Includes 12th grade with no diploma) | 10.02 (8.58–11.67) | 10.92 (9.25–12.86) |  |
| High school graduate/GED or equivalent | 24.89 (22.21–27.79) | 21.13 (18.32–24.24) |  |
| Some college or AA degree | 36.39 (33.43–39.47) | 29.65 (26.57–32.93) |  |
| PIR |  |  | <0.0001 |
| Low | 23.30 (21.02–25.75) | 18.51 (16.10–21.18) |  |
| Middle | 37.04 (34.63–39.51) | 33.44 (30.24–36.81) |  |
| High | 39.66 (36.29–43.14) | 48.05 (43.88–52.24) |  |
| Marital status |  |  | <0.0001 |
| Never married | 7.12 (5.57–9.06) | 7.58 (5.82–9.81) |  |
| Married | 51.98 (49.05–54.89) | 70.69 (67.12–74.03) |  |
| Widowed | 19.87 (17.67–22.28) | 4.63 (3.59–5.96) |  |
| Divorced | 14.63 (12.89–16.55) | 10.73 (8.47–13.50) |  |
| Separated | 2.41 (1.87–3.11) | 1.67 (1.03–2.70) |  |
| Living with partner | 3.99 (3.13–5.08) | 4.69 (3.29–6.64) |  |
| Hypertension |  |  | 0.6695 |
| No | 44.67 (41.98–47.39) | 43.76 (40.10–47.49) |  |
| Yes | 55.33 (52.61–58.02) | 56.24 (52.51–59.90) |  |
| Diabetes |  |  | 0.1238 |
| No | 77.66 (75.38–79.80) | 75.00 (72.02–77.77) |  |
| Yes | 22.34 (20.20–24.62) | 25.00 (22.23–27.98) |  |
| PreCVD |  |  | 0.0362 |
| No | 80.38 (78.03–82.53) | 76.91 (74.07–79.52) |  |
| Yes | 19.62 (17.47–21.97) | 23.09 (20.48–25.93) |  |
| Smoking status |  |  | <0.0001 |
| Never | 51.97 (48.88–55.04) | 35.09 (30.99–39.42) |  |
| Former | 29.84 (26.95–32.90) | 42.63 (38.92–46.43) |  |
| Now | 18.19 (15.85–20.78) | 22.28 (19.26–25.62) |  |
| Drinking status |  |  | <0.0001 |
| Never | 16.07 (14.19–18.14) | 6.69 (5.27–8.46) |  |
| Former | 13.89 (12.33–15.62) | 10.95 (8.83–13.50) |  |
| Mild | 40.34 (37.71–43.03) | 52.70 (48.44–56.92) |  |
| Moderate | 18.89 (16.58–21.44) | 11.67 (9.44–14.35) |  |
| Severe | 10.80 (9.08–12.81) | 17.98 (15.02–21.38) |  |

For continuous variables: survey-weighted mean (95% CI), P-value was by survey-weighted linear regression

For categorical variables: survey-weighted percentage (95% CI), P-value was by survey-weighted Chi-square test
