# Supplementary material for: Do serum vitamins, carotenoids, and retinyl esters influence mortality in osteoarthritis? Insights from a nationally representative study
Source: Front Nutr. 2025 Jun 19;12:1609759. doi: 10.3389/fnut.2025.1609759 (PMC12224656; doi:10.3389/fnut.2025.1609759)
Supplement: Supplementary Figure 1A — Flow chart (vitamin C). [file Data_Sheet_1.zip › Data Sheet 1 (2)/Supplementary Table 2A.docx]

Supplementary Table S2A Cox regression analysis of serum vitamins, carotenoids, and retinyl esters and their non-significant associations with all-cause mortality in OA patients

| All-cause mortality | | | | | | |
| --- | --- | --- | --- | --- | --- | --- |
|  | Model 1 | | Model 2 | | Model 3 | |
| Character | HR (95%CI) | *p* | HR (95%CI) | *p* | HR (95%CI) | *p* |
| Vitamin A | 1.01  (1.0040–1.0161) | 0.0011 | 0.999  (0.9900–1.0081) | 0.8270 | 1.0003  (0.9911–1.0096) | 0.9459 |
| Vitamin C | 1.1419  (0.8685–1.5013) | 0.3420 | 0.72  (0.5509–0.9410) | 0.0162 | 0.9074  (0.6705–1.2280) | 0.5290 |
| Vitamin E | 1.0003  (1.0001–1.0005) | 0.0011 | 1.00  (0.9998–1.0002) | 0.9987 | 1.00  (0.9998–1.0002) | 0.8405 |
| α-carotene | 1.0119  (0.9794–1.0455) | 0.4778 | 0.9525  (0.9007–1.0073) | 0.0879 | 0.9979  (0.9559–1.0419) | 0.9247 |
| Trans-β carotene | 1.0055  (0.9995–1.0116) | 0.0720 | 0.9991  (0.9908–1.0075) | 0.8339 | 1.0052  (0.9994–1.0109) | 0.0783 |
| Cis-β  carotene | 1.0836  (0.9694–1.2112) | 0.1577 | 0.9890  (0.8744–1.1187) | 0.8608 | 1.0811  (0.9834–1.1886) | 0.1067 |
| β-Cryptoxanthin | 0.9855  (0.9599–1.0119) | 0.2793 | 0.9617  (0.9330–0.9914) | 0.0118 | 0.9753  (0.9458–1.0057) | 0.1104 |
| Lutein and zeaxanthin | 1.0116  (0.9982–1.0252) | 0.0913 | 0.985  (0.9683–1.0019) | 0.0816 | 0.9946  (0.9782–1.0114) | 0.5275 |
| Trans-Lycopene | 0.9656  (0.9488–0.9827) | <0.0001 | 0.9879  (0.9712–1.0049) | 0.1615 | 0.999  (0.9831–1.0152) | 0.9056 |

Model 1: No adjustment for covariates. Model 2: Adjusted for age, gender, and race. Model 3: Age, BMI, waist circumference, ALT, AST, race, education level, PIR, marital status, hypertension, diabetes, PreCVD, smoking status, and drinking status.
