# Supplementary material for: Do serum vitamins, carotenoids, and retinyl esters influence mortality in osteoarthritis? Insights from a nationally representative study
Source: Front Nutr. 2025 Jun 19;12:1609759. doi: 10.3389/fnut.2025.1609759 (PMC12224656; doi:10.3389/fnut.2025.1609759)
Supplement: Supplementary Figure 1A — Flow chart (vitamin C). [file Data_Sheet_1.zip › Data Sheet 1 (2)/Supplementary Table 2B.DOCX]

Supplementary Table S2B Cox regression analysis of serum vitamins, carotenoids, and retinyl esters and their non-significant associations with cardiovascular mortality in OA patients

|  | Cardiovascular disease mortality | | | | | |
| --- | --- | --- | --- | --- | --- | --- |
|  | Model 1 | | Model 2 | | Model 3 | |
| Character | HR (95%CI) | *p* | HR (95%CI) | *p* | HR (95%CI) | *p* |
| Vitamin A | 1.0108  (0.9996–1.0220) | 0.0583 | 0.999  (0.9821–1.0162) | 0.9102 | 0.9999  (0.9841–1.0161) | 0.9934 |
| Vitamin D | 0.9947  (0.9873–1.0021) | 0.1584 | 0.9882  (0.9800–0.9964) | 0.0049 | 0.9931  (0.9852–1.0010) | 0.0865 |
| Vitamin E | 1.0003  (1.0000–1.0005) | 0.0386 | 1.00  (0.9996–1.0004) | 0.9720 | 1.0001  (0.9996–1.0005) | 0.8179 |
| α-carotene | 0.9848  (0.9229–1.0509) | 0.6442 | 0.9234  (0.8408–1.0141) | 0.0954 | 0.9608  (0.8872–1.0405) | 0.3250 |
| Trans-β carotene | 0.997  (0.9872–1.0070) | 0.5555 | 0.9787  (0.9590–0.9989) | 0.0385 | 0.9913  (0.9729–1.0101) | 0.3628 |
| Cis-β  carotene | 0.9455  (0.8149–1.0970) | 0.4599 | 0.7523  (0.5860–0.9657) | 0.0255 | 0.9098  (0.7246–1.1424) | 0.4157 |
| β-Cryptoxanthin | 0.9548  (0.9087–1.0033) | 0.0674 | 0.9272  (0.8778–0.9794) | 0.0069 | 0.9444  (0.8848–1.0080) | 0.0852 |
| Lutein and zeaxanthin | 1.0196  (1.0012–1.0383) | 0.0363 | 1.0019  (0.9791–1.0252) | 0.8722 | 1.0007  (0.9806–1.0211) | 0.9479 |
| Trans-Lycopene | 0.9737  (0.9374–1.0113) | 0.1681 | 0.994  (0.9627–1.0264) | 0.7128 | 0.9974  (0.9669–1.0288) | 0.8693 |
| Retinyl Palmitate | 1.056  (0.8896–1.2536) | 0.5335 | 0.8577  (0.5206–1.4131) | 0.5468 | 0.8583  (0.4807–1.5323) | 0.6053 |
| Retinyl  Stearate | 1.0069  (0.9627–1.0531) | 0.7642 | 0.9483  (0.8047–1.1176) | 0.5266 | 0.9168  (0.7780–1.0804) | 0.2999 |

Model 1: No adjustment for covariates. Model 2: Adjusted for age, gender, and race. Model 3: Age, BMI, waist circumference, ALT, AST, race, education level, PIR, marital status, hypertension, diabetes, PreCVD, smoking status, and drinking status.
