# Supplementary material for: Do serum vitamins, carotenoids, and retinyl esters influence mortality in osteoarthritis? Insights from a nationally representative study
Source: Front Nutr. 2025 Jun 19;12:1609759. doi: 10.3389/fnut.2025.1609759 (PMC12224656; doi:10.3389/fnut.2025.1609759)
Supplement: Supplementary Figure 1A — Flow chart (vitamin C). [file Data_Sheet_1.zip › Data Sheet 1 (2)/Supplementary Table 2C.DOCX]

Supplementary Table S2C Cox regression analysis of serum vitamins, carotenoids, and retinyl esters and their non-significant associations with cancer mortality in OA patients

|  | Cancer Diseases mortality | | | | | |
| --- | --- | --- | --- | --- | --- | --- |
|  | Model 1 | | Model 2 | | Model 3 | |
| Character | HR (95%CI) | *p* | HR (95%CI) | *p* | HR (95%CI) | *p* |
| Vitamin A | 1.0034  (0.9865–1.0205) | 0.6945 | 0.9911  (0.9682–1.0144) | 0.4496 | 0.9955  (0.9700–1.0216) | 0.7323 |
| Vitamin C | 0.6665  (0.3794–1.1709) | 0.1582 | 0.399  (0.2176–0.7317) | 0.0030 | 0.631  (0.3041–1.3091) | 0.2162 |
| Vitamin D | 0.9997  (0.9921–1.0073) | 0.9313 | 0.9947  (0.9861–1.0034) | 0.2308 | 0.9957  (0.9879–1.0036) | 0.2890 |
| Vitamin E | 1.0002  (0.9999–1.0005) | 0.1536 | 0.9999  (0.9995–1.0004) | 0.7127 | 0.9999  (0.9994–1.0003) | 0.5343 |
| α-carotene | 0.9521  (0.8568–1.0579) | 0.3611 | 0.8721  (0.7518–1.0117) | 0.0709 | 0.9389  (0.8146–1.0821) | 0.3841 |
| Trans-β carotene | 0.9974  (0.9872–1.0078) | 0.6270 | 0.9811  (0.9595–1.0031) | 0.0911 | 0.9931  (0.9733–1.0133) | 0.4989 |
| Cis-β  carotene | 0.9325  (0.7815–1.1126) | 0.4381 | 0.754  (0.5570–1.0208) | 0.0677 | 0.9142  (0.6808–1.2276) | 0.5508 |
| β-Cryptoxanthin | 0.965  (0.9223–1.0098) | 0.1238 | 0.9387  (0.8943–0.9853) | 0.0105 | 0.966  (0.9188–1.0156) | 0.1755 |
| Lutein and zeaxanthin | 1.0054  (0.9672–1.0451) | 0.7837 | 0.9807  (0.9313–1.0326) | 0.4585 | 1.0048  (0.9607–1.0509) | 0.8357 |
| Trans-Lycopene | 0.9765  (0.9453–1.0088) | 0.1526 | 0.9946  (0.9634–1.0268) | 0.7372 | 1.0039  (0.9649–1.0445) | 0.8469 |

Model 1: No adjustment for covariates. Model 2: Adjusted for age, gender, and race. Model 3: Age, BMI, waist circumference, ALT, AST, race, education level, PIR, marital status, hypertension, diabetes, PreCVD, smoking status, and drinking status.
