# Supplementary material for: Do serum vitamins, carotenoids, and retinyl esters influence mortality in osteoarthritis? Insights from a nationally representative study
Source: Front Nutr. 2025 Jun 19;12:1609759. doi: 10.3389/fnut.2025.1609759 (PMC12224656; doi:10.3389/fnut.2025.1609759)
Supplement: Supplementary Figure 1A — Flow chart (vitamin C). [file Data_Sheet_1.zip › Data Sheet 1 (2)/Supplementary Table 3.DOCX]

**Table S3** Gender-stratified threshold effect analysis of serum vitamins, carotenoids, and retinyl esters and mortality risk in OA patients

|  | Adjusted HR (95% CI), *P* value |
| --- | --- |
| **All-cause mortality** |  |
| **Vitamin D** |  |
| ***Male*** |  |
| Fitting by the standard linear model | 0.99 (0.99–1.00) 0.0012 |
| Fitting by the two-piecewise linear model |  |
| Inflection point | 53.1 |
| < 53.1 (nmol/L) | 0.98 (0.96–0.99) 0.0045 |
| > 53.1 (nmol/L) | 1.00 (0.99–1.00) 0.1921 |
| Log likelihood ratio | 0.069 |
| ***Female*** |  |
| Fitting by the standard linear model | 0.99 (0.99–1.00) 0.0035 |
| Fitting by the two-piecewise linear model |  |
| Inflection point | 30.5 |
| < 30.5 (nmol/L) | 0.93 (0.90–0.96) <0.0001 |
| > 30.5 (nmol/L) | 1.00 (0.99–1.00) 0.1614 |
| Log likelihood ratio | <0.001 |
| **Retinyl Palmitate** |  |
| ***Male*** |  |
| Fitting by the standard linear model | 0.98 (0.88–1.08) 0.6434 |
| Fitting by the two-piecewise linear model |  |
| Inflection point | 1.53 |
| < 1.53 (µg/dL) | 0.65 (0.42–1.01) 0.0561 |
| > 1.53 (µg/dL) | 1.04 (0.93–1.17) 0.4537 |
| Log likelihood ratio | 0.069 |
| ***Female*** |  |
| Fitting by the standard linear model | 0.87 (0.79–0.96) 0.0060 |
| Fitting by the two-piecewise linear model |  |
| Inflection point | 4 |
| < 4 (µg/dL) | 0.97 (0.81–1.16) 0.7175 |
| > 4 (µg/dL) | 0.75 (0.58–0.97) 0.0305 |
| Log likelihood ratio | 0.172 |
| **Cancer Diseases mortality** |  |
| **Retinyl Palmitate** |  |
| ***Male*** |  |
| Fitting by the standard linear model | 0.95 (0.72–1.26) 0.7206 |
| Fitting by the two-piecewise linear model |  |
| Inflection point | 2.9 |
| < 2.9 (µg/dL) | 0.67 (0.37–1.20) 0.1774 |
| > 2.9 (µg/dL) | 1.32 (0.79–2.19) 0.2888 |
| Log likelihood ratio | 0.177 |
| ***Female*** |  |
| Fitting by the standard linear model | 0.74 (0.54–1.01) 0.0598 |
| Fitting by the two-piecewise linear model |  |
| Inflection point | 1.6 |
| < 1.6 (µg/dL) | 0.44 (0.16–1.24) 0.1209 |
| > 1.6 (µg/dL) | 0.84 (0.59–1.18) 0.3080 |
| Log likelihood ratio | 0.301 |

Note: Adjusted for age, BMI, waist circumference, ALT, AST, race, education level, PIR, marital status, hypertension, diabetes, PreCVD, smoking status, and drinking status.
