# Supplementary material for: Do serum vitamins, carotenoids, and retinyl esters influence mortality in osteoarthritis? Insights from a nationally representative study
Source: Front Nutr. 2025 Jun 19;12:1609759. doi: 10.3389/fnut.2025.1609759 (PMC12224656; doi:10.3389/fnut.2025.1609759)
Supplement: Supplementary Figure 1A — Flow chart (vitamin C). [file Data_Sheet_1.zip › Data Sheet 1 (2)/Supplementary Table 4 A to E.DOCX]

Table S4A Subgroup of associations between Retinyl Stearate and all-cause mortality

| Subgroup | Adjusted HR (95% CI) *P* | *P* for interaction |
| --- | --- | --- |
| Drinking status |  | 0.3544 |
| Never | 1.07 (0.57–1.99) 0.8367 |  |
| Former | 1.18 (0.71–1.97) 0.5243 |  |
| Mild | 1.04 (0.82–1.32) 0.7694 |  |
| Moderate | 1.05 (0.89–1.25) 0.5436 |  |
| Severe | 0.73 (0.21–2.58) 0.6282 |  |
| Hypertension |  | 0.6135 |
| No | 1.02 (0.87–1.20) 0.7750 |  |
| Yes | 1.15 (0.86–1.54) 0.3576 |  |
| PIR |  | 0.9231 |
| Low | 0.98 (0.81–1.20) 0.8631 |  |
| Middle | 1.26 (0.96–1.66) 0.0917 |  |
| High | 1.04 (0.76–1.41) 0.8189 |  |
| Gender |  | 0.3296 |
| Female | 1.03 (0.89–1.19) 0.6848 |  |
| Male | 1.07 (0.75–1.52) 0.7027 |  |
| Age (years) |  | 0.6589 |
| < 60 | 0.98 (0.67–1.44) 0.9274 |  |
| ≥ 60 | 1.00 (0.83–1.19) 0.9581 |  |
| Race |  | 0.3145 |
| Other Race - Including Multi-Racial | 0.49 (0.06–3.91) 0.5016 |  |
| Mexican American | 1.63 (1.09–2.46) 0.0187 |  |
| Other Hispanic | 39.61 (0.97–1610.76) 0.0517 |  |
| Non-Hispanic White | 0.97 (0.82–1.15) 0.7201 |  |
| Non-Hispanic Black | 0.81 (0.45–1.46) 0.4865 |  |
| Education level |  | 0.0173 |
| College graduate or above | 0.81 (0.37–1.75) 0.5840 |  |
| Less than 9th grade | 1.45 (1.09–1.92) 0.0107 |  |
| 9-11th grade (Includes 12th grade with no diploma) | 0.51 (0.22–1.17) 0.1141 |  |
| High school graduate/GED or equivalent | 1.26 (0.90–1.77) 0.1723 |  |
| Some college or AA degree | 1.06 (0.90–1.25) 0.4796 |  |
| BMI (kg/m²) |  | 0.0530 |
| Low | 1.32 (1.02–1.70) 0.0348 |  |
| Middle | 0.98 (0.83–1.17) 0.8449 |  |
| High | 0.76 (0.41–1.43) 0.4012 |  |
| Waist circumference (cm) |  | 0.1667 |
| Low | 1.00 (0.84–1.19) 0.9884 |  |
| Middle | 1.45 (1.01–2.09) 0.0466 |  |
| High | 0.98 (0.64–1.51) 0.9419 |  |
| Marital status |  | 0.3975 |
| Never married | 18.85 (1.29–274.53) 0.0317 |  |
| Married | 1.02 (0.66–1.58) 0.9187 |  |
| Widowed | 1.00 (0.84–1.19) 0.9960 |  |
| Divorced | 0.97 (0.45–2.07) 0.9369 |  |
| Separated | 0.38 (0.01–19.63) 0.6274 |  |
| Living with partner | 0.63 (0.03–15.48) 0.7795 |  |
| ALT (U/L) |  | 0.0569 |
| Low | 1.04 (0.62–1.76) 0.8779 |  |
| Middle | 1.06 (0.93–1.21) 0.3749 |  |
| High | 1.01 (0.75–1.35) 0.9598 |  |
| AST (U/L) |  | 0.3789 |
| Low | 1.58 (1.10–2.29) 0.0145 |  |
| Middle | 1.01 (0.84–1.22) 0.9007 |  |
| High | 0.97 (0.76–1.24) 0.8336 |  |
| Smoking status |  | 0.8154 |
| Never | 1.35 (1.02–1.79) 0.0360 |  |
| Former | 0.90 (0.72–1.14) 0.3906 |  |
| Now | 1.40 (0.96–2.03) 0.0799 |  |
| PreCVD |  | 0.6721 |
| No | 0.99 (0.83–1.18) 0.9408 |  |
| Yes | 1.34 (0.95–1.91) 0.1001 |  |
| Diabetes |  | 0.4209 |
| No | 0.96 (0.79–1.17) 0.6704 |  |
| Yes | 1.27 (0.98–1.66) 0.0754 |  |

All adjustment variables except the variables themselves were adjusted as above.

Table S4B Subgroup of associations between Retinyl Palmitate and all-cause mortality

| Subgroup | Adjusted HR (95% CI) *P* | *P* for interaction |
| --- | --- | --- |
| Drinking status |  | 0.6593 |
| Never | 0.92 (0.78–1.09) 0.3438 |  |
| Former | 1.02 (0.88–1.18) 0.7958 |  |
| Mild | 1.00 (0.93–1.08) 0.9192 |  |
| Moderate | 1.00 (0.95–1.05) 0.9445 |  |
| Severe | 1.03 (0.84–1.27) 0.7625 |  |
| Hypertension |  | 0.9070 |
| No | 0.99 (0.95–1.04) 0.8063 |  |
| Yes | 1.00 (0.92–1.09) 0.9806 |  |
| PIR |  | 0.6883 |
| Low | 0.99 (0.93–1.04) 0.6165 |  |
| Middle | 1.05 (0.98–1.14) 0.1846 |  |
| High | 0.94 (0.80–1.10) 0.4328 |  |
| Gender |  | 0.1345 |
| Female | 0.99 (0.95–1.04) 0.7728 |  |
| Male | 1.00 (0.91–1.11) 0.9579 |  |
| Age (years) |  | 0.3587 |
| < 60 | 0.99 (0.89–1.10) 0.8324 |  |
| ≥ 60 | 0.97 (0.91–1.03) 0.2696 |  |
| Race |  | 0.2757 |
| Other Race - Including Multi-Racial | 0.86 (0.52–1.44) 0.5756 |  |
| Mexican American | 1.06 (0.90–1.26) 0.4746 |  |
| Other Hispanic | 1.36 (0.97–1.89) 0.0711 |  |
| Non-Hispanic White | 0.99 (0.94–1.03) 0.5438 |  |
| Non-Hispanic Black | 0.87 (0.71–1.06) 0.1597 |  |
| Education level |  | 0.0753 |
| College graduate or above | 0.90 (0.71–1.16) 0.4264 |  |
| Less than 9th grade | 1.06 (0.95–1.18) 0.2831 |  |
| 9-11th grade (Includes 12th grade with no diploma) | 0.81 (0.65–1.00) 0.0513 |  |
| High school graduate/GED or equivalent | 1.07 (0.98–1.18) 0.1440 |  |
| Some college or AA degree | 1.00 (0.95–1.05) 0.9602 |  |
| BMI (kg/m²) |  | 0.1342 |
| Low | 0.99 (0.89–1.10) 0.8609 |  |
| Middle | 1.00 (0.96–1.04) 0.9148 |  |
| High | 0.93 (0.79–1.09) 0.3844 |  |
| Waist circumference (cm) |  | 0.7736 |
| Low | 0.96 (0.88–1.05) 0.4180 |  |
| Middle | 1.09 (0.99–1.20) 0.0717 |  |
| High | 0.99 (0.88–1.11) 0.8379 |  |
| Marital status |  | 0.7276 |
| Never married | 1.25 (0.75–2.09) 0.3895 |  |
| Married | 1.00 (0.90–1.12) 0.9484 |  |
| Widowed | 0.99 (0.93–1.05) 0.7164 |  |
| Divorced | 0.83 (0.63–1.08) 0.1565 |  |
| Separated | 0.93 (0.64–1.36) 0.7131 |  |
| Living with partner | 0.71 (0.28–1.81) 0.4766 |  |
| ALT (U/L) |  | 0.1961 |
| Low | 1.00 (0.87–1.16) 0.9528 |  |
| Middle | 1.01 (0.97–1.04) 0.7616 |  |
| High | 0.95 (0.85–1.06) 0.3749 |  |
| AST (U/L) |  | 0.2175 |
| Low | 1.10 (0.94–1.28) 0.2252 |  |
| Middle | 1.00 (0.96–1.05) 0.9964 |  |
| High | 0.95 (0.87–1.04) 0.2718 |  |
| Smoking status |  | 0.9850 |
| Never | 1.05 (0.95–1.16) 0.3269 |  |
| Former | 0.96 (0.89–1.04) 0.3151 |  |
| Now | 1.06 (0.94–1.19) 0.3450 |  |
| PreCVD |  | 0.3509 |
| No | 0.97 (0.91–1.04) 0.4411 |  |
| Yes | 1.06 (0.96–1.17) 0.2766 |  |
| Diabetes |  | 0.6412 |
| No | 0.97 (0.91–1.04) 0.4187 |  |
| Yes | 1.04 (0.95–1.13) 0.3815 |  |

All adjustment variables except the variables themselves were adjusted as above.

Table S4C Subgroup of the association between vitamin C and cardiovascular mortality

| Subgroup | Adjusted HR (95% CI) *P* | *P* for interaction |
| --- | --- | --- |
| Drinking status |  | 0.3665 |
| Never | 2.05 (0.96–4.38) 0.0639 |  |
| Former | 1.57 (0.69–3.58) 0.2876 |  |
| Mild | 0.99 (0.47–2.08) 0.9767 |  |
| Moderate | 1.74 (0.49–6.13) 0.3881 |  |
| Severe | 0.69 (0.10–4.88) 0.7128 |  |
| Hypertension |  | 0.6777 |
| No | 2.05 (1.07–3.95) 0.0309 |  |
| Yes | 1.04 (0.60–1.81) 0.8811 |  |
| PIR |  | 0.4797 |
| Low | 1.09 (0.50–2.34) 0.8351 |  |
| Middle | 1.90 (1.02–3.54) 0.0439 |  |
| High | 0.83 (0.27–2.53) 0.7378 |  |
| Gender |  | 0.2075 |
| Female | 1.13 (0.57–2.25) 0.7239 |  |
| Male | 1.68 (1.03–2.74) 0.0361 |  |
| Age (years) |  | 0.4298 |
| < 60 | 1.46 (0.56–3.80) 0.4435 |  |
| ≥ 60 | 1.08 (0.66–1.78) 0.7486 |  |
| Education level |  | 0.0775 |
| College graduate or above | 0.07 (0.00–1.32) 0.0750 |  |
| Less than 9th grade | 0.46 (0.16–1.34) 0.1548 |  |
| 9-11th grade (Includes 12th grade with no diploma) | 1.83 (0.74–4.52) 0.1906 |  |
| High school graduate/GED or equivalent | 3.23 (1.21–8.59) 0.0189 |  |
| Some college or AA degree | 1.76 (1.05–2.95) 0.0325 |  |
| Smoking status |  | 0.2994 |
| Never | 1.63 (0.86–3.11) 0.1352 |  |
| Former | 1.07 (0.50–2.30) 0.8621 |  |
| Now | 1.02 (0.28–3.77) 0.9732 |  |
| PreCVD |  | 0.0975 |
| No | 0.98 (0.52–1.85) 0.9615 |  |
| Yes | 1.77 (1.01–3.09) 0.0457 |  |
| Diabetes |  | 0.5218 |
| No | 1.19 (0.69–2.06) 0.5389 |  |
| Yes | 1.42 (0.74–2.73) 0.2913 |  |

All adjustment variables except the variables themselves were adjusted as above.

Table S4D Subgroup of associations between Retinyl Stearate and cancer mortality

| Subgroup | Adjusted HR (95% CI) *P* | *P* for interaction |
| --- | --- | --- |
| Drinking status |  | 0.0227 |
| Never | 1.16 (0.17–7.73) 0.8770 |  |
| Former | 0.02 (0.00–1.20) 0.0605 |  |
| Mild | 1.00 (0.59–1.71) 0.9875 |  |
| Moderate | 0.08 (0.00–3.90) 0.2057 |  |
| Severe | 3.43 (0.39–30.40) 0.2681 |  |
| Hypertension |  | 0.3980 |
| No | 0.96 (0.59–1.57) 0.8674 |  |
| Yes | 0.67 (0.26–1.72) 0.4000 |  |
| PIR |  | 0.5310 |
| Low | 0.58 (0.12–2.88) 0.5083 |  |
| Middle | 0.97 (0.40–2.34) 0.9453 |  |
| High | 0.93 (0.40–2.16) 0.8591 |  |
| Gender |  | 0.6440 |
| Female | 0.89 (0.49–1.62) 0.6986 |  |
| Male | 0.75 (0.27–2.05) 0.5695 |  |
| Age (years) |  | 0.7312 |
| < 60 | 0.81 (0.15–4.27) 0.8051 |  |
| ≥ 60 | 0.75 (0.39–1.42) 0.3712 |  |
| Education level |  | 0.1422 |
| College graduate or above | 2.01 (0.56–7.17) 0.2839 |  |
| Less than 9th grade | 0.04 (0.00–2.24) 0.1142 |  |
| 9-11th grade (Includes 12th grade with no diploma) | 0.88 (0.36–2.19) 0.7897 |  |
| High school graduate/GED or equivalent | 0.20 (0.02–1.73) 0.1440 |  |
| Some college or AA degree | 0.96 (0.54–1.73) 0.9047 |  |
| BMI (kg/m²) |  | 0.1822 |
| Low | 1.28 (0.62–2.63) 0.5003 |  |
| Middle | 0.80 (0.34–1.88) 0.6126 |  |
| High | 0.30 (0.04–2.23) 0.2403 |  |
| Waist circumference (cm) |  | 0.6024 |
| Low | 0.94 (0.52–1.70) 0.8306 |  |
| Middle | 1.14 (0.43–3.00) 0.7896 |  |
| High | 0.47 (0.12–1.82) 0.2711 |  |
| ALT (U/L) |  | 0.5081 |
| Low | 0.34 (0.03–3.62) 0.3705 |  |
| Middle | 0.83 (0.31–2.25) 0.7126 |  |
| High | 0.96 (0.51–1.80) 0.8953 |  |
| AST (U/L) |  | 0.5380 |
| Low | 0.23 (0.01–8.83) 0.4272 |  |
| Middle | 0.90 (0.42–1.93) 0.7856 |  |
| High | 0.83 (0.41–1.69) 0.6146 |  |
| Smoking status |  | 0.2185 |
| Never | 0.29 (0.03–2.51) 0.2596 |  |
| Former | 0.80 (0.41–1.58) 0.5213 |  |
| Now | 1.37 (0.63–2.98) 0.4252 |  |
| PreCVD |  | 0.2690 |
| No | 0.66 (0.26–1.69) 0.3887 |  |
| Yes | 1.19 (0.47–2.99) 0.7150 |  |
| Diabetes |  | 0.8588 |
| No | 0.76 (0.34–1.69) 0.5029 |  |
| Yes | 1.04 (0.43–2.52) 0.9352 |  |

All adjustment variables except the variables themselves were adjusted as above.

Table S4E Subgroup of associations between Retinyl Palmitate and cancer mortality

| Subgroup | Adjusted HR (95% CI) *P* | *P* for interaction |
| --- | --- | --- |
| Drinking status |  | 0.0136 |
| Never | 0.95 (0.57–1.59) 0.8490 |  |
| Former | 0.61 (0.27–1.40) 0.2462 |  |
| Mild | 1.01 (0.87–1.17) 0.8848 |  |
| Moderate | 0.22 (0.05–1.04) 0.0559 |  |
| Severe | 1.10 (0.67–1.81) 0.7124 |  |
| Hypertension |  | 0.9593 |
| No | 0.94 (0.74–1.21) 0.6415 |  |
| Yes | 0.92 (0.73–1.16) 0.4828 |  |
| PIR |  | 0.2210 |
| Low | 0.86 (0.59–1.26) 0.4464 |  |
| Middle | 1.05 (0.88–1.26) 0.6016 |  |
| High | 0.69 (0.43–1.11) 0.1261 |  |
| Gender |  | 0.3220 |
| Female | 0.92 (0.72–1.17) 0.4795 |  |
| Male | 0.95 (0.74–1.23) 0.7126 |  |
| Age (years) |  | 0.8566 |
| < 60 | 0.86 (0.48–1.52) 0.6013 |  |
| ≥ 60 | 0.89 (0.74–1.08) 0.2390 |  |
| Race |  | 0.6447 |
| Other Race - Including Multi-Racial | 0.61 (0.09–4.04) 0.6069 |  |
| Mexican American | 0.36 (0.12–1.05) 0.0609 |  |
| Other Hispanic | 0.34 (0.01–16.61) 0.5879 |  |
| Non-Hispanic White | 0.98 (0.86–1.11) 0.7140 |  |
| Non-Hispanic Black | 0.81 (0.47–1.39) 0.4496 |  |
| Education level |  | 0.9057 |
| College graduate or above | 1.08 (0.70–1.67) 0.7297 |  |
| Less than 9th grade | 0.86 (0.47–1.54) 0.6036 |  |
| 9-11th grade (Includes 12th grade with no diploma) | 0.91 (0.65–1.26) 0.5589 |  |
| High school graduate/GED or equivalent | 0.88 (0.61–1.27) 0.4825 |  |
| Some college or AA degree | 0.96 (0.73–1.26) 0.7761 |  |
| BMI (kg/m²) |  | 0.5597 |
| Low | 0.87 (0.62–1.20) 0.3912 |  |
| Middle | 0.99 (0.88–1.11) 0.8434 |  |
| High | 0.80 (0.52–1.22) 0.2955 |  |
| Waist circumference (cm) |  | 0.3855 |
| Low | 0.70 (0.41–1.20) 0.1983 |  |
| Middle | 1.09 (0.87–1.37) 0.4483 |  |
| High | 0.86 (0.63–1.18) 0.3492 |  |
| ALT (U/L) |  | 0.3466 |
| Low | 0.66 (0.35–1.26) 0.2076 |  |
| Middle | 0.98 (0.85–1.14) 0.8232 |  |
| High | 0.93 (0.74–1.18) 0.5670 |  |
| AST (U/L) |  | 0.7619 |
| Low | 0.92 (0.52–1.64) 0.7801 |  |
| Middle | 0.98 (0.83–1.15) 0.8160 |  |
| High | 0.88 (0.69–1.12) 0.3017 |  |
| Smoking status |  | 0.8956 |
| Never | 1.03 (0.77–1.38) 0.8417 |  |
| Former | 0.92 (0.73–1.16) 0.4803 |  |
| Now | 0.89 (0.57–1.36) 0.5816 |  |
| PreCVD |  | 0.1441 |
| No | 0.82 (0.62–1.08) 0.1604 |  |
| Yes | 1.07 (0.84–1.36) 0.5935 |  |
| Diabetes |  | 0.9809 |
| No | 0.91 (0.72–1.14) 0.3911 |  |
| Yes | 0.98 (0.76–1.27) 0.8893 |  |

All adjustment variables except the variables themselves were adjusted as above.
