# Supplementary material for: Do serum vitamins, carotenoids, and retinyl esters influence mortality in osteoarthritis? Insights from a nationally representative study
Source: Front Nutr. 2025 Jun 19;12:1609759. doi: 10.3389/fnut.2025.1609759 (PMC12224656; doi:10.3389/fnut.2025.1609759)
Supplement: Supplementary Figure 1A — Flow chart (vitamin C). [file Data_Sheet_1.zip › Data Sheet 1 (2)/Supplementary Table 5 A to C.DOCX]

Supplementary Table S5A Baseline characteristics of the OA patient population based on gender: Excluding the population that died within two years of follow-up

| Variables | Female | Male | *P*-value |
| --- | --- | --- | --- |
| *For continuous variables, mean (95% CI)* | | | |
| Age (years) | 60.21 (58.41–62.01) | 58.77 (57.21–60.33) | 0.2800 |
| BMI (kg/m²) | 30.84 (29.43–32.25) | 30.28 (29.23–31.32) | 0.5705 |
| Waist circumference (cm) | 101.01 (98.19–103.83) | 107.50 (105.13–109.86) | 0.0021 |
| ALT (U/L) | 20.10 (18.81–21.38) | 28.51 (25.37–31.65) | <0.0001 |
| AST (U/L) | 21.57 (20.77–22.36) | 26.37 (24.02–28.72) | 0.0010 |
| Vitamin A (µg/dL) | 59.18 (57.58–60.78) | 65.67 (62.84–68.51) | 0.0003 |
| Vitamin E (µg/dL) | 1454.40 (1387.28–1521.52) | 1309.05 (1250.19–1367.90) | 0.0020 |
| α-carotene (µg/dL) | 4.50 (3.82–5.17) | 3.16 (2.63–3.68) | 0.0033 |
| Trans-β carotene (µg/dL) | 21.26 (17.54–24.98) | 15.08 (12.32–17.83) | 0.0122 |
| Cis-β carotene (µg/dL) | 1.25 (1.06–1.45) | 0.94 (0.76–1.11) | 0.0246 |
| β-Cryptoxanthin (µg/dL) | 8.11 (7.21–9.01) | 7.55 (6.59–8.50) | 0.2844 |
| Lutein and zeaxanthin (µg/dL) | 17.81 (16.21–19.41) | 16.18 (14.72–17.64) | 0.1555 |
| Trans-Lycopene (µg/dL) | 19.50 (18.33–20.67) | 20.26 (18.92–21.59) | 0.3707 |
| Retinyl Palmitate (µg/dL) | 1.93 (1.59–2.27) | 1.88 (1.66–2.10) | 0.7950 |
| Retinyl Stearate (µg/dL) | 0.62 (0.54–0.70) | 0.60 (0.55–0.66) | 0.7392 |
| *For categorical variables, percentage (95% CI)* | | | |
| Race |  |  | 0.0551 |
| Other Race - Including Multi-Racial | 6.81 (4.50–10.19) | 4.21 (2.53–6.93) |  |
| Mexican American | 4.65 (3.34–6.44) | 3.28 (1.91–5.61) |  |
| Other Hispanic | 3.87 (2.38–6.21) | 2.98 (1.32–6.60) |  |
| Non-Hispanic White | 70.90 (65.17–76.04) | 79.55 (72.80–84.97) |  |
| Non-Hispanic Black | 13.76 (10.33–18.10) | 9.98 (7.24–13.60) |  |
| Education level |  |  | 0.0208 |
| College graduate or above | 17.31 (13.36–22.13) | 27.43 (21.04–34.90) |  |
| Less than 9th grade | 6.01 (4.33–8.28) | 6.65 (4.59–9.55) |  |
| 9-11th grade (Includes 12th grade with no diploma) | 11.50 (9.15–14.35) | 12.68 (9.01–17.55) |  |
| High school graduate/GED or equivalent | 29.02 (23.60–35.11) | 23.50 (18.48–29.40) |  |
| Some college or AA degree | 36.17 (29.85–43.00) | 29.74 (24.07–36.10) |  |
| PIR |  |  | 0.1365 |
| Low | 26.26 (21.71–31.39) | 20.80 (15.64–27.13) |  |
| Middle | 35.10 (29.97–40.60) | 33.45 (26.53–41.16) |  |
| High | 38.64 (32.85–44.76) | 45.75 (36.52–55.27) |  |
| Marital status |  |  | 0.0111 |
| Never married | 6.97 (3.34–13.96) | 7.24 (3.71–13.65) |  |
| Married | 53.87 (47.55–60.07) | 63.80 (55.72–71.16) |  |
| Widowed | 20.54 (15.61–26.55) | 7.62 (4.61–12.33) |  |
| Divorced | 13.37 (9.84–17.92) | 14.11 (9.18–21.08) |  |
| Separated | 2.40 (1.52–3.77) | 1.66 (0.82–3.34) |  |
| Living with partner | 2.85 (1.74–4.63) | 5.57 (2.96–10.24) |  |
| Hypertension |  |  | 0.4556 |
| No | 49.04 (42.54–55.57) | 45.86 (38.77–53.12) |  |
| Yes | 50.96 (44.43–57.46) | 54.14 (46.88–61.23) |  |
| Diabetes |  |  | 0.1210 |
| No | 80.36 (76.71–83.56) | 76.76 (71.12–81.59) |  |
| Yes | 19.64 (16.44–23.29) | 23.24 (18.41–28.88) |  |
| Smoking status |  |  | <0.0001 |
| Never | 53.83 (47.15–60.38) | 25.62 (20.01–32.18) |  |
| Former | 26.30 (22.00–31.10) | 44.16 (38.07–50.43) |  |
| Now | 19.87 (15.03–25.79) | 30.22 (24.87–36.16) |  |
| Drinking status |  |  | <0.0001 |
| Never | 16.25 (12.24–21.25) | 5.38 (2.78–10.16) |  |
| Former | 20.16 (17.11–23.59) | 16.84 (11.70–23.64) |  |
| Mild | 36.88 (30.70–43.52) | 43.36 (35.98–51.06) |  |
| Moderate | 18.32 (14.76–22.51) | 11.27 (7.05–17.53) |  |
| Severe | 8.40 (5.00–13.77) | 23.15 (17.12–30.51) |  |
| PreCVD |  |  | 0.1673 |
| No | 80.60 (76.50–84.14) | 77.10 (72.19–81.36) |  |
| Yes | 19.40 (15.86–23.50) | 22.90 (18.64–27.81) |  |

For continuous variables: survey-weighted mean (95% CI), P-value was by survey-weighted linear regression

For categorical variables: survey-weighted percentage (95% CI), P-value was by survey-weighted Chi-square test

Supplementary Table S5B Baseline characteristics of the OA patient population based on gender and vitamin C: Excluding the population that died within two years of follow-up

| Variables | Female | Male | *P*-value |
| --- | --- | --- | --- |
| *For continuous variables, mean (95% CI)* | | | |
| Age (years) | 61.05 (59.64–62.47) | 59.83 (58.27–61.38) | 0.2729 |
| BMI (kg/m²) | 31.15 (29.82–32.48) | 30.28 (29.45–31.10) | 0.3454 |
| Waist circumference (cm) | 101.96 (99.29–104.64) | 107.57 (105.72–109.41) | 0.0022 |
| ALT (U/L) | 20.04 (18.48–21.60) | 25.61 (22.85–28.38) | 0.0009 |
| AST (U/L) | 21.83 (20.69–22.98) | 24.86 (22.71–27.00) | 0.0239 |
| Vitamin C (mg/dL) | 1.04 (0.97–1.11) | 0.85 (0.79–0.92) | <0.0001 |
| *For categorical variables, percentage (95% CI)* | | | |
| Race |  |  | 0.4762 |
| Other Race - Including Multi-Racial | 6.84 (4.34–10.64) | 8.31 (4.60–14.56) |  |
| Mexican American | 4.54 (3.20–6.41) | 3.11 (1.84–5.21) |  |
| Other Hispanic | 3.51 (2.27–5.39) | 2.07 (0.80–5.28) |  |
| Non-Hispanic White | 73.96 (68.54–78.75) | 78.06 (70.92–83.84) |  |
| Non-Hispanic Black | 11.14 (8.39–14.65) | 8.44 (6.02–11.72) |  |
| Education level |  |  | 0.0251 |
| College graduate or above | 20.64 (16.34–25.73) | 31.02 (24.37–38.55) |  |
| Less than 9th grade | 4.31 (3.00–6.15) | 4.50 (2.81–7.12) |  |
| 9-11th grade (Includes 12th grade with no diploma) | 8.16 (6.36–10.40) | 10.57 (7.68–14.38) |  |
| High school graduate/GED or equivalent | 31.46 (26.28–37.14) | 25.17 (19.89–31.29) |  |
| Some college or AA degree | 35.44 (28.92–42.55) | 28.75 (23.31–34.88) |  |
| PIR |  |  | 0.1632 |
| Low | 22.91 (18.64–27.83) | 16.66 (11.99–22.69) |  |
| Middle | 37.65 (32.46–43.14) | 39.06 (33.32–45.12) |  |
| High | 39.44 (33.32–45.91) | 44.28 (37.15–51.64) |  |
| Marital status |  |  | 0.0020 |
| Never married | 7.75 (4.56–12.87) | 6.20 (3.12–11.94) |  |
| Married | 52.53 (46.73–58.26) | 67.90 (60.26–74.68) |  |
| Widowed | 19.78 (14.74–26.03) | 6.80 (4.22–10.78) |  |
| Divorced | 13.77 (9.96–18.75) | 12.83 (8.18–19.55) |  |
| Separated | 2.07 (1.30–3.29) | 1.42 (0.68–2.93) |  |
| Living with partner | 4.09 (2.51–6.60) | 4.85 (2.66–8.71) |  |
| Hypertension |  |  | 0.3664 |
| No | 46.03 (40.42–51.75) | 42.30 (35.27–49.65) |  |
| Yes | 53.97 (48.25–59.58) | 57.70 (50.35–64.73) |  |
| Diabetes |  |  | 0.8330 |
| No | 77.15 (71.90–81.67) | 77.76 (72.84–82.00) |  |
| Yes | 22.85 (18.33–28.10) | 22.24 (18.00–27.16) |  |
| Smoking status |  |  | <0.0001 |
| Never | 54.80 (48.09–61.33) | 27.90 (22.19–34.42) |  |
| Former | 27.32 (22.85–32.29) | 48.33 (41.97–54.74) |  |
| Now | 17.89 (13.91–22.70) | 23.78 (18.69–29.74) |  |
| Drinking status |  |  | 0.0001 |
| Never | 13.04 (9.47–17.70) | 5.01 (2.84–8.69) |  |
| Former | 20.58 (17.42–24.16) | 20.75 (14.82–28.26) |  |
| Mild | 37.55 (32.33–43.07) | 38.91 (32.99–45.19) |  |
| Moderate | 18.49 (14.89–22.71) | 11.78 (7.04–19.06) |  |
| Severe | 10.34 (6.52–16.02) | 23.55 (17.15–31.43) |  |
| PreCVD |  |  | 0.6740 |
| No | 78.26 (72.23–83.28) | 76.87 (71.25–81.67) |  |
| Yes | 21.74 (16.72–27.77) | 23.13 (18.33–28.75) |  |

For continuous variables: survey-weighted mean (95% CI), P-value was by survey-weighted linear regression

For categorical variables: survey-weighted percentage (95% CI), P-value was by survey-weighted Chi-square test

Supplementary Table S5C Baseline characteristics of the OA patient population based on gender and vitamin D: Excluding the population that died within two years of follow-up

| Variables | Female | Male | *P*-value |
| --- | --- | --- | --- |
| *For continuous variables, mean (95% CI)* | | | |
| Age (years) | 61.15 (60.43–61.87) | 59.46 (58.56–60.35) | 0.0068 |
| BMI (kg/m²) | 31.24 (30.67–31.81) | 30.52 (30.09–30.95) | 0.0543 |
| Waist circumference (cm) | 102.29 (101.17–103.40) | 107.89 (106.87–108.90) | <0.0001 |
| ALT (U/L) | 21.48 (20.73–22.22) | 27.68 (26.47–28.89) | <0.0001 |
| AST (U/L) | 23.74 (23.15–24.33) | 26.48 (25.50–27.46) | <0.0001 |
| Vitamin D (nmol/L) | 80.47 (78.08–82.86) | 74.52 (72.47–76.57) | 0.0001 |
| *For categorical variables, percentage (95% CI)* | | | |
| Race |  |  | 0.0992 |
| Other Race - Including Multi-Racial | 5.32 (4.21–6.71) | 6.37 (4.58–8.79) |  |
| Mexican American | 3.88 (3.09–4.87) | 3.62 (2.67–4.89) |  |
| Other Hispanic | 3.40 (2.68–4.30) | 2.83 (1.98–4.03) |  |
| Non-Hispanic White | 77.30 (74.71–79.69) | 79.51 (76.19–82.47) |  |
| Non-Hispanic Black | 10.11 (8.59–11.86) | 7.68 (6.30–9.32) |  |
| Education level |  |  | <0.0001 |
| College graduate or above | 23.79 (21.15–26.66) | 33.37 (29.33–37.67) |  |
| Less than 9th grade | 5.19 (4.25–6.32) | 5.05 (3.99–6.37) |  |
| 9-11th grade (Includes 12th grade with no diploma) | 9.88 (8.44–11.54) | 10.80 (9.08–12.81) |  |
| High school graduate/GED or equivalent | 24.77 (22.10–27.65) | 20.60 (17.63–23.93) |  |
| Some college or AA degree | 36.37 (33.39–39.46) | 30.18 (27.04–33.51) |  |
| PIR |  |  | <0.0001 |
| Low | 23.09 (20.78–25.58) | 18.38 (15.86–21.20) |  |
| Middle | 36.86 (34.42–39.37) | 33.26 (30.03–36.66) |  |
| High | 40.05 (36.65–43.54) | 48.36 (44.15–52.59) |  |
| Marital status |  |  | <0.0001 |
| Never married | 7.28 (5.70–9.26) | 7.71 (5.89–10.04) |  |
| Married | 52.71 (49.72–55.68) | 71.07 (67.33–74.54) |  |
| Widowed | 18.90 (16.69–21.32) | 4.20 (3.13–5.60) |  |
| Divorced | 14.69 (12.88–16.70) | 10.60 (8.32–13.43) |  |
| Separated | 2.47 (1.91–3.19) | 1.72 (1.07–2.78) |  |
| Living with partner | 3.96 (3.10–5.04) | 4.69 (3.27–6.68) |  |
| Hypertension |  |  | 0.4860 |
| No | 45.44 (42.72–48.18) | 43.96 (40.43–47.55) |  |
| Yes | 54.56 (51.82–57.28) | 56.04 (52.45–59.57) |  |
| Diabetes |  |  | 0.0501 |
| No | 78.20 (75.91–80.33) | 74.96 (71.90–77.79) |  |
| Yes | 21.80 (19.67–24.09) | 25.04 (22.21–28.10) |  |
| Smoking status |  |  | <0.0001 |
| Never | 52.08 (48.97–55.18) | 34.96 (30.87–39.28) |  |
| Former | 29.73 (26.81–32.83) | 42.62 (38.94–46.39) |  |
| Now | 18.19 (15.84–20.80) | 22.42 (19.28–25.90) |  |
| Drinking status |  |  | <0.0001 |
| Never | 15.93 (14.04–18.02) | 6.46 (5.00–8.30) |  |
| Former | 13.64 (12.09–15.34) | 10.82 (8.66–13.45) |  |
| Mild | 40.43 (37.76–43.16) | 52.67 (48.34–56.96) |  |
| Moderate | 19.12 (16.79–21.70) | 11.90 (9.61–14.64) |  |
| Severe | 10.88 (9.13–12.92) | 18.16 (15.13–21.63) |  |
| PreCVD |  |  | 0.0161 |
| No | 81.30 (78.93–83.47) | 77.24 (74.36–79.88) |  |
| Yes | 18.70 (16.53–21.07) | 22.76 (20.12–25.64) |  |

For continuous variables: survey-weighted mean (95% CI), P-value was by survey-weighted linear regression

For categorical variables: survey-weighted percentage (95% CI), P-value was by survey-weighted Chi-square test
