# Supplementary material for: Do serum vitamins, carotenoids, and retinyl esters influence mortality in osteoarthritis? Insights from a nationally representative study
Source: Front Nutr. 2025 Jun 19;12:1609759. doi: 10.3389/fnut.2025.1609759 (PMC12224656; doi:10.3389/fnut.2025.1609759)
Supplement: Supplementary Figure 1A — Flow chart (vitamin C). [file Data_Sheet_1.zip › Data Sheet 1 (2)/Supplementary Table 6 A to C.DOCX]

Supplementary Table S6A Baseline characteristics of the OA patient population based on gender: Pre CVD population was excluded

| Variables | Female | Male | *P*-value |
| --- | --- | --- | --- |
| *For continuous variables, mean (95% CI)* | | | |
| Age (years) | 59.18 (57.14–61.22) | 56.77 (54.97–58.56) | 0.1202 |
| BMI (kg/m²) | 30.97 (29.29–32.64) | 30.39 (29.23–31.55) | 0.6128 |
| Waist circumference (cm) | 100.94 (97.57–104.30) | 107.67 (104.93–110.40) | 0.0045 |
| ALT (U/L) | 19.90 (18.70–21.11) | 29.51 (26.18–32.85) | <0.0001 |
| AST (U/L) | 21.48 (20.56–22.41) | 27.13 (24.47–29.79) | 0.0006 |
| Vitamin A (µg/dL) | 58.68 (57.02–60.34) | 63.89 (60.55–67.23) | 0.0083 |
| Vitamin E (µg/dL) | 1448.44 (1373.34–1523.54) | 1297.70 (1232.04–1363.35) | 0.0028 |
| α-carotene (µg/dL) | 4.56 (3.85–5.27) | 3.15 (2.59–3.72) | 0.0002 |
| Trans-β carotene (µg/dL) | 22.11 (17.64–26.58) | 14.92 (11.56–18.27) | 0.0189 |
| Cis-β carotene (µg/dL) | 1.29 (1.06–1.52) | 0.93 (0.72–1.14) | 0.0394 |
| β-Cryptoxanthin (µg/dL) | 8.31 (7.24–9.39) | 7.20 (6.26–8.14) | 0.0864 |
| Lutein and zeaxanthin (µg/dL) | 18.46 (16.55–20.38) | 16.55 (14.71–18.39) | 0.1993 |
| Trans-Lycopene (µg/dL) | 20.13 (18.85–21.41) | 20.62 (19.09–22.15) | 0.6219 |
| Retinyl Palmitate (µg/dL) | 1.86 (1.48–2.25) | 1.83 (1.57–2.09) | 0.8818 |
| Retinyl Stearate (µg/dL) | 0.60 (0.52–0.69) | 0.60 (0.53–0.67) | 0.9683 |
| *For categorical variables, percentage (95% CI)* | | | |
| Race |  |  | 0.0800 |
| Other Race - Including Multi-Racial | 7.00 (4.36–11.03) | 3.99 (2.39–6.61) |  |
| Mexican American | 4.70 (3.24–6.78) | 3.40 (1.83–6.25) |  |
| Other Hispanic | 3.89 (2.27–6.59) | 3.54 (1.54–7.97) |  |
| Non-Hispanic White | 70.79 (64.57–76.31) | 79.10 (71.11–85.33) |  |
| Non-Hispanic Black | 13.62 (10.13–18.07) | 9.96 (6.84–14.29) |  |
| Education level |  |  | 0.0649 |
| College graduate or above | 19.03 (14.54–24.52) | 29.02 (21.44–38.00) |  |
| Less than 9th grade | 5.04 (3.31–7.60) | 5.48 (3.22–9.16) |  |
| 9-11th grade (Includes 12th grade with no diploma) | 10.00 (7.73–12.86) | 11.75 (7.80–17.32) |  |
| High school graduate/GED or equivalent | 28.34 (22.80–34.61) | 24.66 (18.67–31.81) |  |
| Some college or AA degree | 37.59 (30.55–45.18) | 29.10 (22.25–37.05) |  |
| PIR |  |  | 0.2769 |
| Low | 25.08 (19.85–31.14) | 21.18 (15.91–27.63) |  |
| Middle | 34.07 (29.00–39.55) | 30.94 (23.30–39.78) |  |
| High | 40.85 (34.58–47.43) | 47.87 (37.22–58.73) |  |
| Marital status |  |  | 0.0072 |
| Never married | 7.18 (3.02–16.14) | 7.76 (3.30–17.15) |  |
| Married | 54.07 (47.07–60.90) | 64.41 (54.77–73.01) |  |
| Widowed | 19.42 (14.17–26.01) | 4.83 (2.02–11.07) |  |
| Divorced | 13.62 (9.47–19.19) | 14.87 (8.95–23.69) |  |
| Separated | 2.81 (1.75–4.46) | 1.04 (0.44–2.41) |  |
| Living with partner | 2.91 (1.66–5.07) | 7.10 (3.79–12.90) |  |
| Hypertension |  |  | 0.5552 |
| No | 53.67 (47.01–60.20) | 51.02 (41.93–60.05) |  |
| Yes | 46.33 (39.80–52.99) | 48.98 (39.95–58.07) |  |
| Diabetes |  |  | 0.8879 |
| No | 83.37 (79.14–86.88) | 82.89 (75.28–88.51) |  |
| Yes | 16.63 (13.12–20.86) | 17.11 (11.49–24.72) |  |
| Smoking status |  |  | <0.0001 |
| Never | 54.20 (46.57–61.65) | 27.98 (21.00–36.22) |  |
| Former | 26.59 (21.38–32.54) | 41.30 (34.29–48.67) |  |
| Now | 19.21 (14.24–25.40) | 30.72 (24.37–37.90) |  |
| Drinking status |  |  | 0.0001 |
| Never | 15.31 (11.00–20.92) | 4.72 (1.80–11.80) |  |
| Former | 18.50 (14.93–22.71) | 17.13 (11.46–24.82) |  |
| Mild | 38.47 (31.22–46.28) | 41.06 (31.97–50.81) |  |
| Moderate | 19.11 (14.97–24.08) | 11.45 (6.55–19.25) |  |
| Severe | 8.60 (4.65–15.34) | 25.64 (18.19–34.84) |  |

For continuous variables: survey-weighted mean (95% CI), P-value was by survey-weighted linear regression

For categorical variables: survey-weighted percentage (95% CI), P-value was by survey-weighted Chi-square test

Supplementary Table S6B Baseline characteristics of the OA patient population based on gender and vitamin C: Pre CVD population was excluded

| Variables | Female | Male | *P*-value |
| --- | --- | --- | --- |
| *For continuous variables, mean (95% CI)* | | | |
| Age (years) | 59.79 (58.08–61.49) | 58.10 (56.25–59.94) | 0.2381 |
| BMI (kg/m²) | 30.97 (29.33–32.62) | 30.12 (29.25–31.00) | 0.4298 |
| Waist circumference (cm) | 101.31 (98.16–104.46) | 107.23 (105.21–109.24) | 0.0054 |
| ALT (U/L) | 20.18 (18.72–21.63) | 26.52 (23.71–29.33) | 0.0005 |
| AST (U/L) | 22.17 (20.79–23.55) | 25.53 (23.25–27.81) | 0.0293 |
| Vitamin C (mg/dL) | 1.05 (0.98–1.12) | 0.85 (0.78–0.92) | <0.0001 |
| *For categorical variables, percentage (95% CI)* | | | |
| Race |  |  | 0.4298 |
| Other Race - Including Multi-Racial | 7.56 (4.50–12.43) | 6.21 (3.34–11.26) |  |
| Mexican American | 4.88 (3.32–7.12) | 3.56 (2.04–6.15) |  |
| Other Hispanic | 3.52 (2.14–5.75) | 2.45 (0.91–6.43) |  |
| Non-Hispanic White | 72.30 (65.37–78.31) | 79.78 (72.24–85.67) |  |
| Non-Hispanic Black | 11.73 (8.53–15.93) | 8.00 (5.59–11.34) |  |
| Education level |  |  | 0.0931 |
| College graduate or above | 22.28 (17.50–27.92) | 31.29 (24.53–38.96) |  |
| Less than 9th grade | 4.01 (2.61–6.11) | 4.19 (2.22–7.76) |  |
| 9-11th grade (Includes 12th grade with no diploma) | 7.48 (5.50–10.12) | 10.17 (6.95–14.65) |  |
| High school graduate/GED or equivalent | 29.39 (24.23–35.13) | 25.71 (20.20–32.11) |  |
| Some college or AA degree | 36.84 (29.87–44.41) | 28.64 (22.14–36.17) |  |
| PIR |  |  | 0.1486 |
| Low | 22.76 (17.59–28.92) | 15.62 (11.35–21.11) |  |
| Middle | 36.34 (31.46–41.52) | 37.19 (30.57–44.33) |  |
| High | 40.90 (33.89–48.30) | 47.19 (39.14–55.39) |  |
| Marital status |  |  | 0.0055 |
| Never married | 8.50 (4.58–15.25) | 6.86 (3.03–14.77) |  |
| Married | 53.33 (46.73–59.82) | 68.19 (59.56–75.74) |  |
| Widowed | 18.14 (13.09–24.58) | 4.67 (2.15–9.85) |  |
| Divorced | 13.49 (9.25–19.26) | 13.97 (8.18–22.84) |  |
| Separated | 2.67 (1.69–4.18) | 0.92 (0.39–2.14) |  |
| Living with partner | 3.87 (1.90–7.75) | 5.39 (3.01–9.47) |  |
| Hypertension |  |  | 0.4762 |
| No | 51.84 (46.27–57.36) | 47.96 (38.14–57.94) |  |
| Yes | 48.16 (42.64–53.73) | 52.04 (42.06–61.86) |  |
| Diabetes |  |  | 0.5614 |
| No | 82.32 (77.19–86.50) | 84.05 (76.92–89.28) |  |
| Yes | 17.68 (13.50–22.81) | 15.95 (10.72–23.08) |  |
| Smoking status |  |  | <0.0001 |
| Never | 55.12 (48.13–61.92) | 30.44 (22.77–39.38) |  |
| Former | 27.20 (22.67–32.26) | 45.39 (37.67–53.33) |  |
| Now | 17.67 (13.30–23.10) | 24.17 (18.61–30.75) |  |
| Drinking status |  |  | 0.0002 |
| Never | 13.77 (9.76–19.09) | 4.88 (2.55–9.16) |  |
| Former | 19.40 (16.03–23.28) | 17.94 (12.57–24.94) |  |
| Mild | 38.06 (31.69–44.87) | 37.68 (30.73–45.17) |  |
| Moderate | 18.22 (14.21–23.04) | 13.31 (7.75–21.93) |  |
| Severe | 10.55 (6.19–17.40) | 26.19 (18.34–35.92) |  |

For continuous variables: survey-weighted mean (95% CI), P-value was by survey-weighted linear regression

For categorical variables: survey-weighted percentage (95% CI), P-value was by survey-weighted Chi-square test

Supplementary Table S6C Baseline characteristics of the OA patient population based on gender and vitamin D: Pre CVD population was excluded

| Variables | Female | Male | *P*-value |
| --- | --- | --- | --- |
| *For continuous variables, mean (95% CI)* | | | |
| Age (years) | 60.26 (59.41–61.11) | 57.60 (56.55–58.64) | 0.0003 |
| BMI (kg/m²) | 30.99 (30.34–31.63) | 30.39 (29.88–30.90) | 0.1512 |
| Waist circumference (cm) | 101.66 (100.39–102.93) | 107.46 (106.25–108.68) | <0.0001 |
| ALT (U/L) | 21.70 (20.78–22.63) | 28.39 (27.05–29.72) | <0.0001 |
| AST (U/L) | 24.19 (23.11–25.27) | 26.93 (25.80–28.06) | 0.0006 |
| Vitamin D (nmol/L) | 81.09 (78.38–83.80) | 74.11 (72.15–76.06) | <0.0001 |
| *For categorical variables, percentage (95% CI)* | | | |
| Race |  |  | 0.3697 |
| Other Race - Including Multi-Racial | 5.35 (4.15–6.86) | 4.97 (3.57–6.89) |  |
| Mexican American | 3.92 (3.08–4.99) | 4.21 (3.06–5.76) |  |
| Other Hispanic | 3.45 (2.66–4.44) | 3.10 (2.10–4.54) |  |
| Non-Hispanic White | 77.70 (74.86–80.29) | 80.15 (76.76–83.15) |  |
| Non-Hispanic Black | 9.59 (7.97–11.49) | 7.57 (6.16–9.27) |  |
| Education level |  |  | 0.0012 |
| College graduate or above | 26.63 (23.64–29.85) | 35.27 (30.65–40.18) |  |
| Less than 9th grade | 4.47 (3.51–5.68) | 4.11 (3.02–5.58) |  |
| 9-11th grade (Includes 12th grade with no diploma) | 9.04 (7.58–10.74) | 10.12 (8.22–12.41) |  |
| High school graduate/GED or equivalent | 23.77 (20.94–26.85) | 20.15 (16.88–23.87) |  |
| Some college or AA degree | 36.10 (33.05–39.27) | 30.34 (26.59–34.38) |  |
| PIR |  |  | 0.0001 |
| Low | 21.50 (18.92–24.33) | 17.88 (15.35–20.71) |  |
| Middle | 35.32 (32.45–38.30) | 29.22 (25.78–32.90) |  |
| High | 43.17 (39.15–47.30) | 52.91 (48.37–57.40) |  |
| Marital status |  |  | <0.0001 |
| Never married | 7.46 (5.63–9.84) | 8.59 (6.35–11.53) |  |
| Married | 54.43 (51.22–57.61) | 71.43 (67.19–75.32) |  |
| Widowed | 16.92 (14.78–19.31) | 3.27 (2.23–4.78) |  |
| Divorced | 14.86 (12.90–17.06) | 10.52 (7.81–14.02) |  |
| Separated | 2.68 (2.05–3.51) | 1.71 (0.97–3.01) |  |
| Living with partner | 3.64 (2.62–5.03) | 4.48 (3.00–6.64) |  |
| Hypertension |  |  | 0.4173 |
| No | 50.29 (47.30–53.27) | 48.15 (43.42–52.92) |  |
| Yes | 49.71 (46.73–52.70) | 51.85 (47.08–56.58) |  |
| Diabetes |  |  | 0.2659 |
| No | 81.74 (79.59–83.72) | 79.93 (76.47–83.00) |  |
| Yes | 18.26 (16.28–20.41) | 20.07 (17.00–23.53) |  |
| Smoking status |  |  | <0.0001 |
| Never | 53.07 (49.50–56.61) | 38.02 (33.35–42.93) |  |
| Former | 29.59 (26.22–33.19) | 39.61 (35.39–43.99) |  |
| Now | 17.34 (14.82–20.20) | 22.36 (19.15–25.94) |  |
| Drinking status |  |  | <0.0001 |
| Never | 15.21 (13.14–17.53) | 6.26 (4.64–8.39) |  |
| Former | 12.26 (10.62–14.13) | 8.90 (6.97–11.32) |  |
| Mild | 41.19 (38.04–44.41) | 51.90 (47.50–56.27) |  |
| Moderate | 20.11 (17.55–22.95) | 13.06 (10.35–16.35) |  |
| Severe | 11.23 (9.27–13.54) | 19.88 (16.20–24.15) |  |

For continuous variables: survey-weighted mean (95% CI), P-value was by survey-weighted linear regression

For categorical variables: survey-weighted percentage (95% CI), P-value was by survey-weighted Chi-square test
