# Supplementary material for: Do serum vitamins, carotenoids, and retinyl esters influence mortality in osteoarthritis? Insights from a nationally representative study
Source: Front Nutr. 2025 Jun 19;12:1609759. doi: 10.3389/fnut.2025.1609759 (PMC12224656; doi:10.3389/fnut.2025.1609759)
Supplement: Supplementary Figure 1A — Flow chart (vitamin C). [file Data_Sheet_1.zip › Data Sheet 1 (2)/Supplementary Table 7A.DOCX]

Table S7A Cox regression analysis of serum vitamin and carotenoid levels in relation to mortality risk among OA patients: Excluding the population that died within two years of follow-up

| All-cause mortality | | | | | | |
| --- | --- | --- | --- | --- | --- | --- |
|  | Model 1 | | Model 2 | | Model 3 | |
| Character | HR (95%CI) | *p* | HR (95%CI) | *p* | HR (95%CI) | *p* |
| Vitamin D | 0.998  (0.9935–1.0025) | 0.3721 | 0.9913  (0.9865–0.9962) | 0.0005 | 0.9938  (0.9888–0.9989) | 0.0162 |
| Retinyl Palmitate | 1.0009  (0.9688–1.0341) | 0.9564 | 0.9088  (0.8213–1.0057) | 0.0643 | 0.8866  (0.8281–0.9493) | 0.0005 |
| Retinyl Stearate | 1.0357  (0.9110–1.1775) | 0.5919 | 0.7689  (0.5700–1.0371) | 0.0852 | 0.6744  (0.4995–0.9105) | 0.0101 |
| Cardiovascular disease mortality | | | | | | |
|  | Model 1 | | Model 2 | | Model 3 | |
| Character | HR (95%CI) | *p* | HR (95%CI) | *p* | HR (95%CI) | *p* |
| Vitamin C | 0.8156  (0.7659–1.9629) | 0.3958 | 0.9135  (0.4338–1.9240) | 0.8119 | 1.8393  (0.7651–4.4219) | 0.1733 |
| Cancer Diseases mortality | | | | | | |
|  | Model 1 | | Model 2 | | Model 3 | |
| Character | HR (95%CI) | *p* | HR (95%CI) | *p* | HR (95%CI) | *p* |
| Retinyl Palmitate | 0.9797  (0.9226–1.0403) | 0.5034 | 0.8599  (0.7429–0.9953) | 0.0431 | 0.852  (0.6871–1.0566) | 0.1446 |
| Retinyl Stearate | 0.9447  (0.7868–1.1344) | 0.5423 | 0.6103  (0.3442–1.0822) | 0.0911 | 0.5152  (0.2495–1.0638) | 0.0730 |

Model 1: No adjustment for covariates. Model 2: Adjusted for age, gender, and race. Model 3: Age, BMI, waist circumference, ALT, AST, race, education level, PIR, marital status, hypertension, diabetes, PreCVD, smoking status, and drinking status.
