# Supplementary material for: Do serum vitamins, carotenoids, and retinyl esters influence mortality in osteoarthritis? Insights from a nationally representative study
Source: Front Nutr. 2025 Jun 19;12:1609759. doi: 10.3389/fnut.2025.1609759 (PMC12224656; doi:10.3389/fnut.2025.1609759)
Supplement: Supplementary Figure 1A — Flow chart (vitamin C). [file Data_Sheet_1.zip › Data Sheet 1 (2)/Supplementary Table 7B.DOCX]

Table S7B Cox regression analysis of serum vitamin and carotenoid levels in relation to mortality risk among OA patients: Pre CVD population was excluded

| All-cause mortality | | | | | | |
| --- | --- | --- | --- | --- | --- | --- |
|  | Model 1 | | Model 2 | | Model 3 | |
| Character | HR (95%CI) | *p* | HR (95%CI) | *p* | HR (95%CI) | *p* |
| Vitamin D | 0.9995  (0.9951–1.0040) | 0.8411 | 0.9933  (0.9883–0.9983) | 0.0090 | 0.9949  (0.9901–0.9997) | 0.0374 |
| Retinyl Palmitate | 0.9847  (0.9439–1.0271) | 0.4732 | 0.8931  (0.7759–1.0279) | 0.1149 | 0.911  (0.8326–0.9968) | 0.0424 |
| Retinyl Stearate | 0.9777  (0.8301–1.1517) | 0.7875 | 0.735  (0.4784–1.1290) | 0.1597 | 0.7607  (0.5084–1.1382) | 0.1834 |
| Cardiovascular disease mortality | | | | | | |
|  | Model 1 | | Model 2 | | Model 3 | |
| Character | HR (95%CI) | *p* | HR (95%CI) | *p* | HR (95%CI) | *p* |
| Vitamin C | 1.2274  (0.8017–1.8792) | 0.3458 | 1.0444  (0.4897–2.2274) | 0.9105 | 1.7054  (0.8961–3.2455) | 0.1040 |
| Cancer Diseases mortality | | | | | | |
|  | Model 1 | | Model 2 | | Model 3 | |
| Character | HR (95%CI) | *p* | HR (95%CI) | *p* | HR (95%CI) | *p* |
| Retinyl Palmitate | 0.8935  (0.6999–1.1407) | 0.3662 | 0.7478  (0.5363–1.0427) | 0.0866 | 0.7851  (0.5817–1.0596) | 0.1138 |
| Retinyl Stearate | 0.8729  (0.5950–1.2804) | 0.4866 | 0.5715  (0.2389–1.3671) | 0.2086 | 0.5603  (0.2612–1.2015) | 0.1367 |

Model 1: No adjustment for covariates. Model 2: Adjusted for age, gender, and race. Model 3: Age, BMI, waist circumference, ALT, AST, race, education level, PIR, marital status, hypertension, diabetes, smoking status, and drinking status.
