# Supplementary material for: Do serum vitamins, carotenoids, and retinyl esters influence mortality in osteoarthritis? Insights from a nationally representative study
Source: Front Nutr. 2025 Jun 19;12:1609759. doi: 10.3389/fnut.2025.1609759 (PMC12224656; doi:10.3389/fnut.2025.1609759)
Supplement: Supplementary Figure 1A — Flow chart (vitamin C). [file Data_Sheet_1.zip › Data Sheet 1 (2)/Supplementary Table 8.DOCX]

Table S8 Cox regression analysis of serum vitamin and carotenoid levels and risk of death in patients without OA

| All-cause mortality | | | | | | |
| --- | --- | --- | --- | --- | --- | --- |
|  | Model 1 | | Model 2 | | Model 3 | |
| Character | HR (95%CI) | *p* | HR (95%CI) | *p* | HR (95%CI) | *p* |
| Vitamin D | 0.996  (0.9941–0.9980) | <0.0001 | 0.9906  (0.9888–0.9924) | <0.0001 | 0.994  (0.9923–0.9957) | <0.0001 |
| Retinyl Palmitate | 1.034  (1.0200–1.0481) | <0.0001 | 0.9902  (0.9688–1.0120) | 0.3746 | 1.0084  (0.9903–1.0267) | 0.3662 |
| Retinyl Stearate | 1.1712  (1.1249–1.2193) | <0.0001 | 1.02  (0.9712–1.0713) | 0.4277 | 1.0414  (0.9939–1.0912) | 0.0888 |
| Cardiovascular disease mortality | | | | | | |
|  | Model 1 | | Model 2 | | Model 3 | |
| Character | HR (95%CI) | *p* | HR (95%CI) | *p* | HR (95%CI) | *p* |
| Vitamin C | 1.1805  (0.9733–1.4318) | 0.0920 | 0.6228  (0.5136–0.7553) | <0.0001 | 0.8834  (0.7429–1.0504) | 0.1605 |
| Cancer Diseases mortality | | | | | | |
|  | Model 1 | | Model 2 | | Model 3 | |
| Character | HR (95%CI) | *p* | HR (95%CI) | *p* | HR (95%CI) | *p* |
| Retinyl Palmitate | 1.0366  (1.0111–1.0627) | 0.0047 | 1.0001  (0.9582–1.0437) | 0.9976 | 1.018  (0.9846–1.0525) | 0.2948 |
| Retinyl Stearate | 1.1583  (1.1059–1.2131) | <0.0001 | 1.0057  (0.8674–1.1660) | 0.9402 | 1.0376  (0.9232–1.1663) | 0.5355 |

Model 1: No adjustment for covariates. Model 2: Adjusted for age, gender, and race. Model 3: Age, BMI, waist circumference, ALT, AST, race, education level, PIR, marital status, hypertension, diabetes, PreCVD, smoking status, and drinking status.
