# Supplementary figures and images for: Do serum vitamins, carotenoids, and retinyl esters influence mortality in osteoarthritis? Insights from a nationally representative study
Source: Front Nutr. 2025 Jun 19;12:1609759. doi: 10.3389/fnut.2025.1609759 (PMC12224656; doi:10.3389/fnut.2025.1609759)

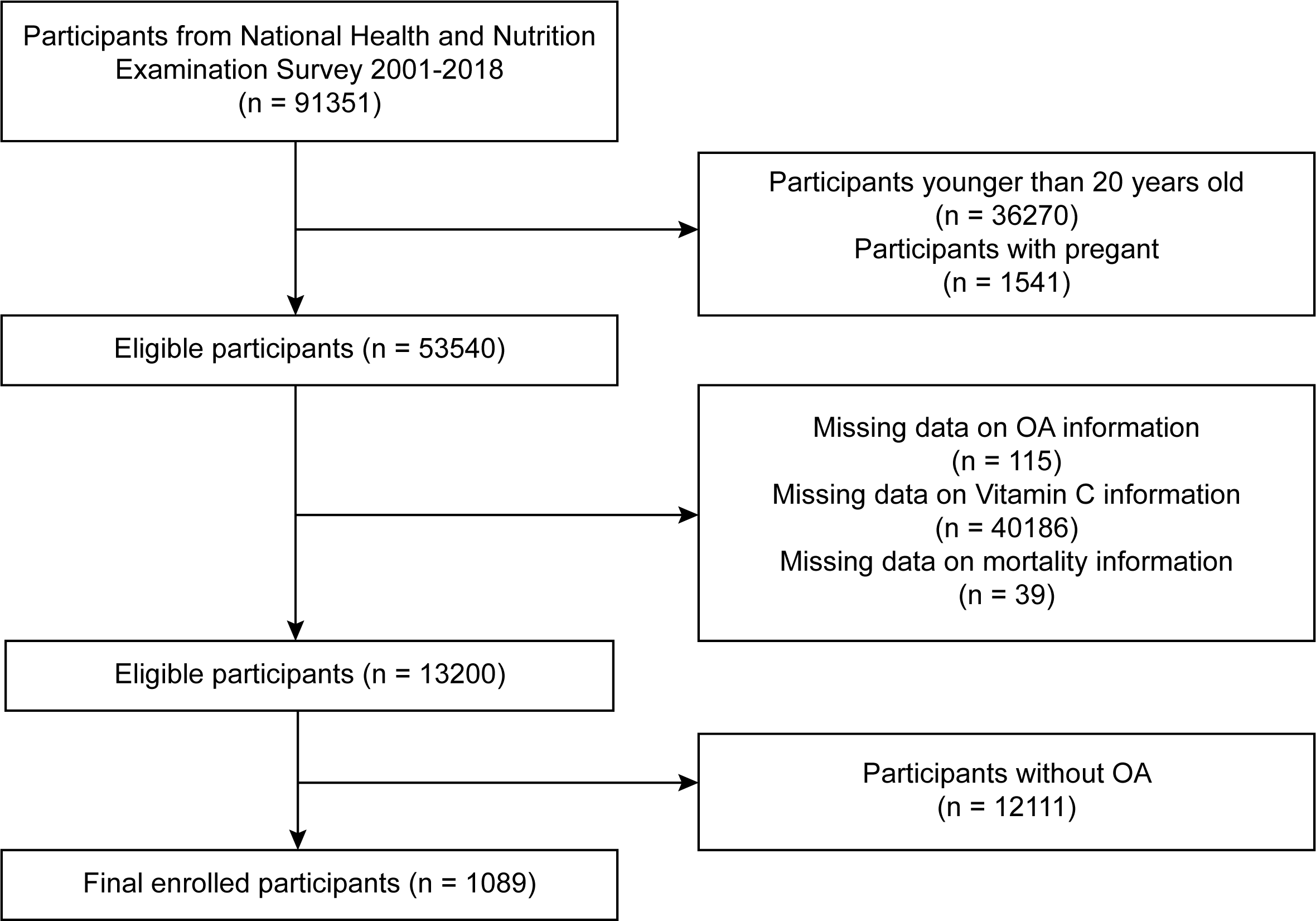

Supplement: Supplementary Figure 1A — Flow chart (vitamin C). [file Data_Sheet_1.zip › Data Sheet 1 (2)/Supplementary Image 1.png]

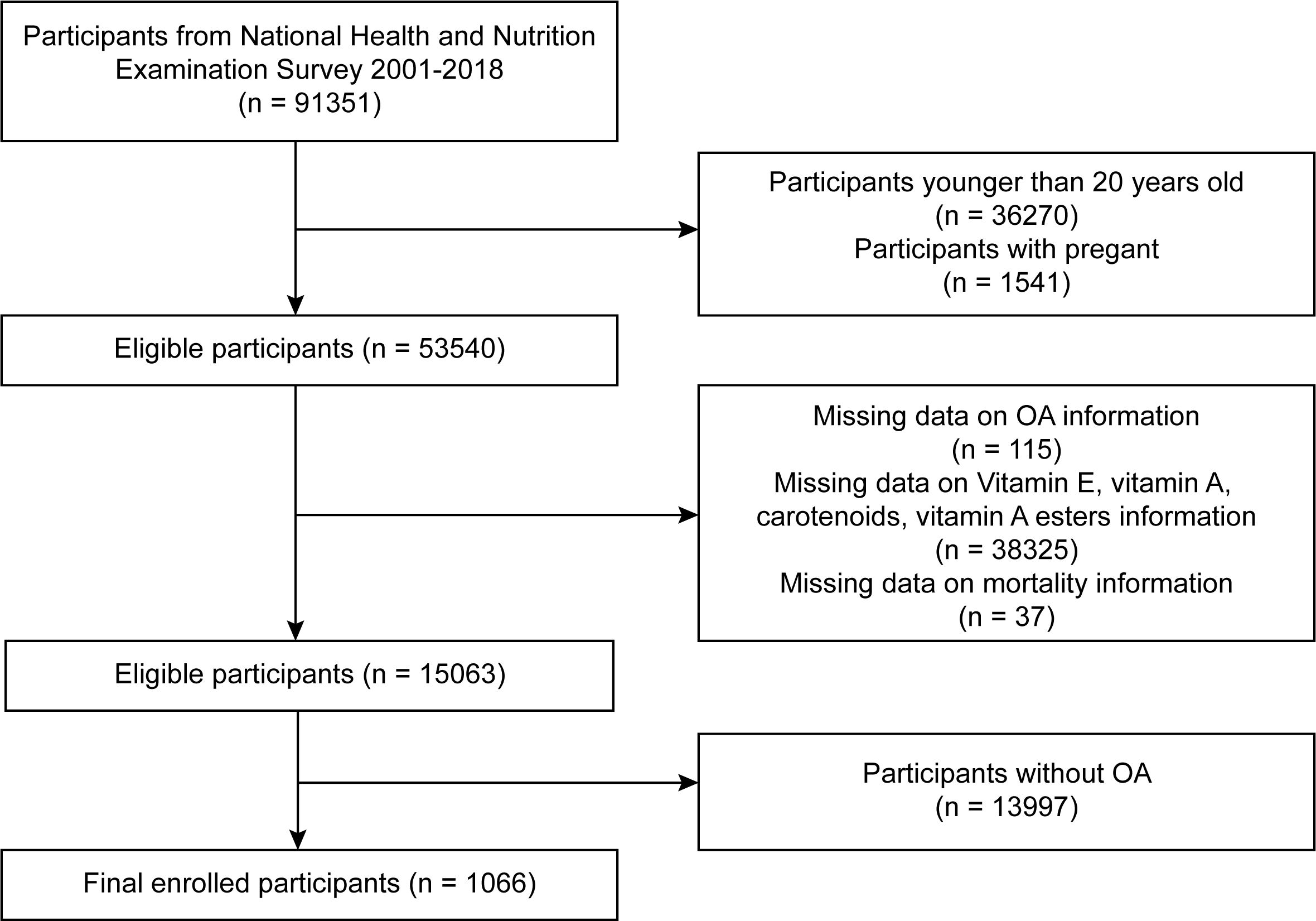

Supplement: Supplementary Figure 1A — Flow chart (vitamin C). [file Data_Sheet_1.zip › Data Sheet 1 (2)/Supplementary Image 2.png]
